# Comparison of Treatment Retention of Adults With Opioid Addiction Managed With Extended-Release Buprenorphine vs Daily Sublingual Buprenorphine-Naloxone at Time of Release From Jail

**DOI:** 10.1001/jamanetworkopen.2021.23032

**Published:** 2021-09-08

**Authors:** Joshua D. Lee, Mia Malone, Ryan McDonald, Anna Cheng, Kumar Vasudevan, Babak Tofighi, Ann Garment, Barbara Porter, Keith S. Goldfeld, Michael Matteo, Jasdeep Mangat, Monica Katyal, Jonathan Giftos, Ross MacDonald

**Affiliations:** 1 Department of Population Health, New York University Grossman School of Medicine, New York; 2 Department of Medicine, New York University Grossman School of Medicine, New York; 3 Correctional Health Services, NYC Health + Hospitals, New York, New York

## Abstract

**Importance:**

Extended-release buprenorphine (XRB), a monthly injectable long-acting opioid use disorder (OUD) treatment, has not been studied for use in corrections facilities.

**Objective:**

To compare treatment retention following release from jail among adults receiving daily sublingual buprenorphine-naloxone (SLB) vs those receiving XRB.

**Design, Setting, and Participants:**

This open-label, randomized comparative effectiveness study included 52 incarcerated adults in New York City observed for 8 weeks postrelease between June 2019 and May 2020. Participants were soon-to-be-released volunteers from 1 men’s and 1 women’s jail facility who had OUDs already treated with SLB. Follow-up treatment was received at a primary care clinic in Manhattan. Data were analyzed between June 2020 and December 2020.

**Interventions:**

XRB treatment was offered prior to release and continued monthly through 8 weeks after release. SLB participants continued to receive daily directly observed in-jail SLB administration, were provided a 7-day SLB supply at jail release, and followed up at a designated clinic (or other preferred clinics).

**Main Outcomes and Measures:**

Buprenorphine treatment retention at 8 weeks postrelease.

**Results:**

A total of 52 participants were randomized 1:1 to XRB (26 participants) and SLB (26 participants). Participants had a mean (SD) age of 42.6 (10.0) years; 45 participants (87%) were men; and 40 (77%) primarily used heroin prior to incarceration. Most participants (30 [58%]) reported prior buprenorphine use; 18 (35%) reported active community buprenorphine treatment prior to jail admission. Twenty-one of 26 assigned to XRB received 1 or more XRB injection prior to release; 3 initiated XRB postrelease; and 2 did not receive XRB. Patients in the XRB arm had fewer jail medical visits compared with daily SLB medication administration (mean [SD] visits per day: XRB, 0.11 [0.03] vs SLB, 1.06 [0.08]). Community buprenorphine treatment retention at week 8 postrelease was 18 participants in the XRB group (69.2%) vs 9 in the SLB group (34.6%), and rates of opioid-negative urine tests were 72 of 130 tests in the XRB group (55.3%) and 50 of 130 tests in the SLB group (38.4%). There were no differences in rates of serious adverse events, no overdoses, and no deaths.

**Conclusions and Relevance:**

XRB was acceptable among patients currently receiving SLB, and patients had fewer in-jail clinic visits and increased community buprenorphine treatment retention when compared with standard daily SLB treatment. These results support wider use and further study of XRB as correctional and reentry OUD treatment.

**Trial Registration:**

ClinicalTrials.gov Identifier: NCT03604159

## Key Points

**Question:**

Is extended-release buprenorphine an acceptable and helpful jail-to-community treatment option among adults already receiving daily sublingual buprenorphine for opioid use disorders?

**Findings:**

In this pilot proof-of-concept comparative effectiveness study of 52 incarcerated adults with opioid use disorders, extended-release buprenorphine was acceptable to most participants, making it a feasible option in the setting of a large jail opioid treatment program; community buprenorphine treatment retention at 8 weeks postrelease was relatively high compared with sublingual buprenorphine.

**Meaning:**

These results suggest that monthly injectable extended-release buprenorphine was a useful opioid use disorder treatment option before and after release from jail.

## Teaser

This comparative effectiveness study assesses the effectiveness of extended-release buprenorphine injections to treat opioid use disorder among adults transitioning from jail to the community compared with daily sublingual buprenorphine treatment.

## Introduction

Opioids were involved in 49 860 (70.6%) of all overdose deaths in 2019, and these rates are likely to have continued to rise in 2020 and 2021.^1,2^ Rates of opioid use in populations involved in the criminal justice system are disproportionately high, as are overdoses following release from jail or prison.^3,4,5,6^ When medications for opioid use disorder (MOUD) are accessible during and after incarceration, treatment retention improves and postrelease opioid relapse, overdose, and mortality are likely to decline.^7,8,9^ Despite these positive public health outcomes, the availability of MOUD in US correctional facilities, including thousands of local municipal jails, has been rare.^10^

New York City jails have offered methadone detoxification and maintenance treatments since the 1980s and currently prescribe methadone, buprenorphine, and naltrexone OUD treatments in the general adult jail pretrial detention and sentenced populations.^9,11,12,13^ A clinical trial conducted in a NYC jail population between 2006 and 2008^14^ estimated 48% of individuals who initiated daily sublingual buprenorphine in-jail successfully linked to community buprenorphine treatment immediately after release. Internal data (from J.M.) indicates recent and pre-COVID rates of postrelease community buprenorphine treatment in NYC were around 50%. Sublingual buprenorphine-naloxone (SLB) treatment in NYC jails is provided daily using directly observed therapy (DOT) dosing in a medical clinic. This is done to prevent diversion and is similar to DOT methadone and other in-jail controlled substance administration.

Injectable extended-release buprenorphine (XRB) (Indivior) is a monthly μ opioid receptor partial agonist, approved for the treatment of opioid use disorders in the US.^15^ XRB is administered monthly in a premixed subcutaneous formulation equivalent to a 16 to 24 mg/d maintenance dose of sublingual buprenorphine-naloxone (SLB). XRB has not been rigorously studied in US correctional settings and represents a new MOUD option for patients on or interested in buprenorphine maintenance. XRB avoids daily DOT protocols and there is no immediate risk of diversion. The long-acting preparation produces a 4-week or longer sustained buprenorphine blood level that increases monthly with subsequent doses, which reduced heroin use during treatment compared with placebo in a double-masked industry phase III pivotal trial.^16^

This proof-of-concept comparative effectiveness study sought to pilot the administration of XRB within the large and well-established NYC jail opioid treatment program and examine its acceptability and feasibility. We compared XRB with usual care SLB among adult volunteers who were receiving SLB for OUD and had an upcoming release date. The principal comparative effectiveness outcome was postrelease community buprenorphine treatment retention. We hypothesized XRB would be acceptable (ie, the majority assigned to XRB would receive an initial injection in jail and a second postrelease), be feasible for the opioid treatment program (OTP) to administer, and have higher rates of community buprenorphine retention.

## Methods

### Study Design

This was a pilot proof-of-concept, open-label, nonmasked, 8-week randomized comparative effectiveness trial of XRB vs SLB among adults with opioid use disorder currently maintained on SLB and incarcerated in NYC jails with upcoming release dates (Supplement 1). The primary study outcome was a description of XRB’s overall acceptability and feasibility. The main comparative effectiveness outcome was community buprenorphine treatment retention at 8 weeks postrelease.

### Study Population, Ethics, and Sites

This study recruited adults diagnosed with OUD incarcerated in NYC Department of Correction–managed jails. Eligible participants: (1) were over age 18 years, (2) met *Diagnostic and Statistical Manual of Mental Disorders* (Fifth Edition) (*DSM-5*) criteria for opioid use disorder, (3) were incarcerated in a NYC jail with a known release date, (4) were currently prescribed daily SLB, (5) were without serious, uncontrolled medical or psychiatric illnesses, and (6) were able to understand and provide written informed consent in English. The only criterion for exclusion was pregnancy or plans for conception. This study was approved by the NYU Grossman School of Medicine institutional review board and met Office for Human Research Protection criteria for federally funded research among prisoners. This report follows the Consolidated Standards of Reporting Trials (CONSORT) reporting guideline.

Recruitment took place from June 2019 through December 2019 with final community follow-up in May 2020. During the study period, the NYC jail daily census counted between 6300 and 7300 detained and incarcerated persons across 10 facilities, 8 of which are located on Rikers Island (Bronx, NY). Correctional Health Services (CHS) of NYC Health + Hospitals operates the nation’s oldest and largest jail-based opioid treatment program, which administers to the 10% to 16% of the adult jail population with OUDs. Participants were recruited from the Eric M. Taylor Center (a sentenced-inmate facility for men) and the Rose M. Singer Center (the lone facility for women in the system). During the study period, the jail opioid treatment program treated approximately 100 to 200 patients daily with SLB and 500 to 800 patients daily with methadone.

**Figure 1.**
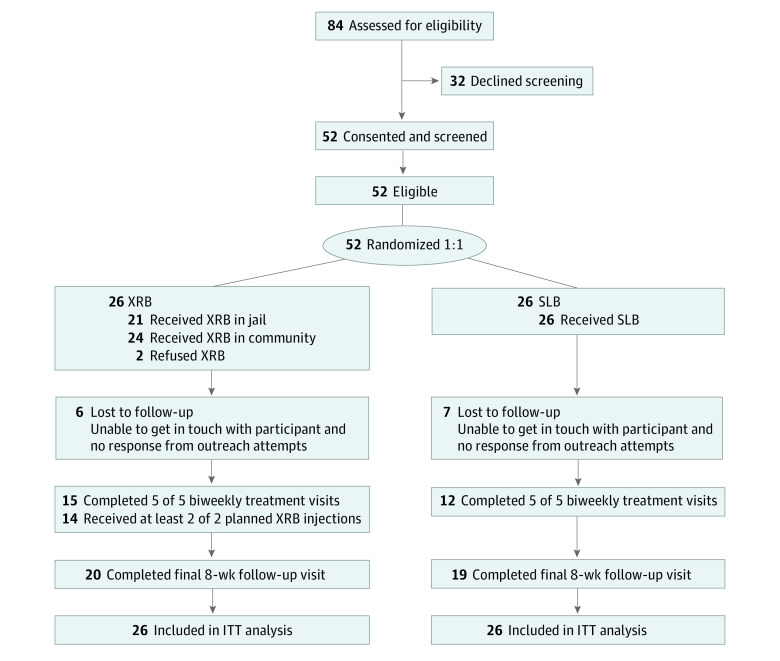
Participant Flowchart

Study and program staff used electronic medical records and program rosters to identify and detail potential participants. Written informed consent preceded screening, eligibility was confirmed with study clinicians, and randomization and XRB induction occurred within 1 to 12 weeks of scheduled release. A total of 52 individuals consented and were screened and randomized (26 to the XRB arm and 26 to the SLB arm) (Figure 1). Current SLB patients declining study screening (32 of 84 approached [38%]) often stated they preferred to remain on SLB.

Postrelease research follow-up visits (weeks 1, 2, 4, 6, and 8) occurred at Bellevue Hospital Center in Manhattan, by telephone if in-person attendance was not possible, or, in the event of reincarceration, at the same jail medical clinics used for the initial treatment. Participants were compensated after release for each completed visit, up to $320: $25 for screening, $25 for participation in the randomization stage (paid at first community visit), $50 per follow-up visit (weeks 1, 2, 4, 6), and $70 for week 8 final research visit.

### Random Assignment

A random allocation sequence in permuted blocks (3:3) and stratified by gender was generated by an independent biostatistician. Study staff opened sealed envelopes within 12 weeks of scheduled release. The study treatment arm was nonmasked and without placebo.

### Study Medication and Intervention

XRB participants were offered the initial XRB monthly injection at least 1 week prior to release. Multiple prerelease XRB doses were available if the release date was 1 or more months away or changed.

CHS medical and nursing staff were trained and supervised by the OTP medical directors (J.G., J.M.). This included screening a manufacturer-produced informational video on the medication and new pharmacy and nursing procedures. XRB doses were refrigerated and stored in secure pharmacy space, then delivered by a pharmacist to the clinical staff just prior to a planned injection. XRB was tightly controlled to ensure minimal risk of loss, diversion, or self-injection. XRB used in jail treatment centers was purchased by CHS directly from usual distributors after registering with the Risk Evaluation and Mitigation Strategy program in accordance with policies for use of commercial products, and jail medication costs were reimbursed by the study.

Postrelease XRB injections were administered at Bellevue free-of-charge using in-kind donations of the study drug. Per the product label, the XRB dosing schedule started at 300 mg monthly for 2 months followed by 100 mg monthly thereafter. Study clinicians had the flexibility to continue the 300 mg dose for longer than 2 months if the participant’s opioid use history or clinical status warranted. XRB participants could decline initial or further XRB treatment and cross back to SLB at any time before or after release. At 8 weeks postrelease, all XRB and SLB participants wishing to continue or initiate XRB treatment were offered continued in-kind XRB treatment in an open-label therapeutic extension study through week 28.

SLB participants remained on SLB in the form of buprenorphine-naloxone tablets (Zubsolv) through daily in-clinic nurse-led DOT visits. Per NYC jail standard of care, SLB was physically provided to each participant as a 7-day take-home supply upon release, which was intended to bridge SLB treatment until a community prescription could be filled. SLB participants could receive postrelease SLB from the Bellevue adult primary care addiction medicine clinic or choose to follow-up with other preferred community SLB providers.

### Study Assessments and Outcomes

The primary aim was to describe the in-jail and postrelease feasibility and acceptability of XRB using a mixed-methods assessment of overall recruitment and study retention rates, the percentage of participants randomized to the XRB group who received 1 or more dose, jail medical clinic visit rates, diversion and adverse events, and qualitative feedback from participants and clinicians. The main comparative effectiveness outcome was community buprenorphine treatment retention rates through 8 weeks after release. Retention was measured as follows: active prescription for any form of buprenorphine 8 weeks after release, percentage of weeks any form of buprenorphine was prescribed, and percentage of weeks participants were retained on study-assigned buprenorphine formulations (XRB vs SLB). Secondary outcomes of interest were heroin and fentanyl use, adverse events, and NYC jail reincarceration.

Baseline assessments consisted of participant demographics, self-reported drug and alcohol treatment and legal histories, a prearrest recall of drug and alcohol use (using timeline follow-back methods), a medication treatment for OUD exposure and preference form, and CHS Electronic Medical Record medical and psychiatric history and laboratory data, including HIV and hepatitis C virus (HCV) status. Postrelease follow-up assessments included: self-reported opioid and other drug use (using timeline follow-back methods); urine testing for fentanyl, morphine (ie, heroin), oxycodone, buprenorphine, and methadone metabolites; pregnancy testing; and adverse event monitoring. Study XRB medication administration logs, hospital medical records, and NYS Prescription Monitoring Program audits performed by a study author (J.D.L.) provided unmasked buprenorphine pharmacy refill and treatment retention data.

Trained study staff (A.C., M.M.) recorded and transcribed open-ended qualitative interviews with XRB participants postrelease to query individual reentry experiences on XRB. Open coding, grounded theory, and a social cognitive theory framework was used to define themes and hypotheses (A.C., M.M., B.T., J.D.L.). CHS coauthors (J.M., J.G.) commented on the in-jail implementation experience.

### Statistical Analysis

Results were descriptive and avoided statistical testing by arm. We estimated a target sample size of 50 participants would provide adequate practical experience and moderate power for estimated rates of treatment retention. Implementation outcomes were analyzed from descriptive measures (ie, rates of accrual, XRB induction success, in-jail clinic visits, diversion events, staff feedback, and XRB participant interviews). Analysis was conducted with Stata version 14 (StataCorp).

## Results

Participants were primarily men (45 [87%]) using heroin and not in treatment at the time of arrest and incarceration; mean (SD) age was 42.6 (10) years. A total of 18 participants (35%; 7 in the XRB arm and 11 in the SLB arm) reported active community SLB treatment at the time of their arrest (Table 1). Forty participants (77%) reported heroin use prior to incarceration, and 30 (58%) reported prior buprenorphine use.

**Table 1.** Baseline Demographics

Characteristics	Participants, No. (%)	
	XRB (n = 26)	SLB (n = 26)
Men	23 (88)	22 (85)
Age, mean (SD)	43.1 (9.2)	42.3 (10.8)
Race/ethnicity		
Hispanic	14 (54)	9 (35)
Non-Hispanic		
Black	5 (19)	8 (31)
White	5 (19)	5 (19)
Other race/ethnicity^a^	2 (8)	4 (15)
High school graduate or higher	15 (58)	16 (62)
Employed (full- or part-time)	12 (46)	14 (54)
30 d prior to incarceration		
Heroin/opioid use	19 (73)	21 (81)
IV use	8 (31)	6 (23)
Lifetime treatment intake episodes, mean (SD)	3.3 (2.9)	3.2 (2.5)
Tried buprenorphine treatment prior to in-jail program participation	15 (58)	15 (58)
Active community SLB treatment prior to incarceration	7 (27)	11 (42)

Abbreviations: SLB, sublingual buprenorphine; XRB, extended-release buprenorphine.

^a^ Any verbal self-reported non-Hispanic race other than listed categories was classified as other (responses included Italian and Asian).

### Acceptability and XRB Exposure

XRB participants received a mean (SD) 2.3 (1.3) injections total. Most (21 of 26) XRB participants received 1 or more doses prior to release, 3 initiated XRB postrelease, and 2 received no XRB. Reasons for the 5 missed prerelease injections included: misunderstandings about XRB and study procedures (2 participants), interruptions to daily SLB routines, intention to switch to methadone, and unscheduled early release (1 participant apiece). Two participants received 2 XRB injections and 1 participant received 3 injections prior to release because of later release dates. Most XRB participants (16 of 26 [62%]) received the minimum planned 2 monthly injections (1 in-jail, 1 postrelease). At the study’s 8-week end point, 6 participants (23%) in the XRB arm and no participants in the SLB arm enrolled in the open-label XRB extension study.

### Feasibility and In-Jail Events

Jail clinic visits declined markedly among XRB participants: mean (SD) visits per day in the XRB arm were 0.11 (0.03) following initiation vs 1.06 (SD 0.08) visits per day in the SLB arm. There were no reported instances of XRB diversion in-jail (eg, stolen or missing doses, attempted subcutaneous nodule extraction postinjection) vs 2 observed episodes of SLB diversion. No in-jail serious adverse events (SAEs) were observed.

Barriers to XRB in-jail induction included apprehension or lack of knowledge about the novel formulation, opposition to needle sticks, perceived limited community nonstudy XRB access, and preference for the more familiar SLB routines. Facilitators to XRB were no daily DOT attendance and less urgency for follow-up care immediately postrelease. In jail, XRB was significantly time- and labor-saving vs daily SLB in-person administration. A more complete assessment of XRB participant experiences, treatment satisfaction, and reentry dynamics before and after COVID-19 will be summarized in a subsequent manuscript.

### Comparative Effectiveness and Retention in Treatment

Retention in community buprenorphine treatment in weeks 1 through 8 postrelease was as follows: 18 participants (69%) in the XRB arm and 9 participants (35%) in the SLB arm were retained on any form of community buprenorphine treatment at week 8; 15 participants (57%) in the XRB arm and 9 participants (35%) in the SLB arm were retained on the assigned buprenorphine formulation treatment at week 8; mean (SD) total weeks of any form of buprenorphine treatment were 6.1 (3.5) weeks in the XRB arm vs 2.6 (3.2) weeks in the SLB arm (Table 2). Seven XRB participants (27%) chose to switch back to SLB prior to week 8, with reported reasons including complaints of burning and pain during XRB administration and general preferences for daily SLB self-administration.

**Table 2.** Acceptability, Feasibility, and Postrelease Comparative Outcomes

Characteristics	Participants, No. (%)			
	XRB (n = 26)	95% CI	SLB (n = 26)	95% CI
Received assigned study medication	24 (92)	NA	26 (100)	NA
Received assigned study medication prior to release as scheduled	21 (81)	NA	26 (100)	NA
Jail medical clinic visits following study medication induction, mean (SD), visits/d	0.11 (0.03)	0.05-0.16	1.06 (0.08)	0.88-1.23
Retained on any form of community buprenorphine treatment at week 8	18 (69)	50-84	9 (35)	19-54
Retained on assigned treatment at week 8	15 (58)	39-74	9 (35)	19-54
Weeks on buprenorphine treatment, mean (SD)	6.1 (3.5)	4.7-7.5	2.6 (3.2)	1.3-3.9
XRB participants who switched back to SLB postrelease	7 (27)	14-46	NA	NA
Urine samples opioid-negative^a^	72 (55.4)	47-64	50 (38.5)	30-47
Reincarcerated	2 (8)	2-24	4 (15)	6-34

Abbreviations: NA, not applicable; SLB, sublingual buprenorphine; XRB, extended-release buprenorphine.

^a^ A total of 130 samples were collected.

**Figure 2.**
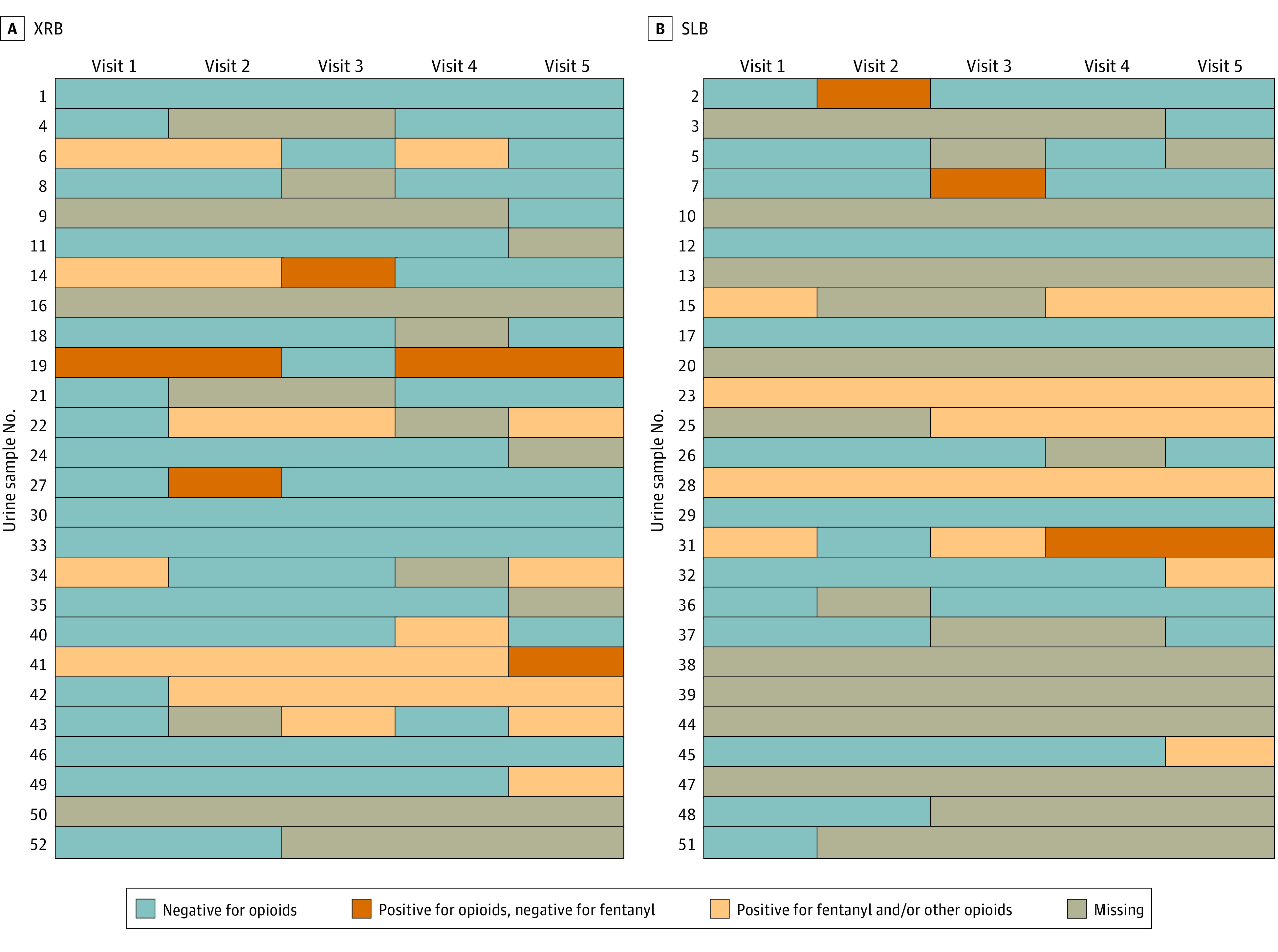
Urine Toxicology Results for Nonprescribed Opioids, Excluding Buprenorphine

Five urine samples per individual participant were scheduled weekly during postrelease weeks 1 through 4 and then at week 8. Missing samples totaled 29 of 130 samples (22.3%) in the XRB group vs 56 of 130 samples (43.1%) in the SLB group. A total of 72 of 130 samples (55.3%) tested negative for any nonprescribed opioids for the XRB arm and 50 of 130 samples (38.4%) tested negative in the SLB arm (Figure 2). Across all 260 samples, opioid-positive results were primarily positive for both morphine (ie, heroin) and fentanyl metabolites (44 [16.9%] morphine [heroin], 38 [14.6%] fentanyl, 9 [3.8%] oxycodone, 4 [1.5%] methadone). Samples positive for fentanyl only were relatively rare (3 samples [1.2%]), as were opioid-positive samples without fentanyl (12 [4.6%]), indicating a high rate of fentanyl-heroin mixing in the NYC heroin supply during 2019 and 2020. Reincarceration in NYC jails occurred for 2 participants (8%) in the XRB arm and 4 (15%) SLB participants. No differences were noted in terms of rates of SAEs (2 participants in the XRB arm vs 0 in the SLB arm), overdoses (0 in each arm), or death (0 in each arm). There were 8 adverse events reported, the most common being tenderness at the XRB injection site and non-study–related emergency department visits. The 2 total SAEs were in the same XRB participant, who developed osteomyelitis and underwent a toe amputation, both likely related to ongoing intravenous cocaine use.

## Discussion

In this proof-of-concept comparative effectiveness study, monthly injectable extended-release buprenorphine appeared acceptable, feasible, and as effective as standard daily sublingual buprenorphine treatment before and after release from jail. More XRB participants (69% vs 35%) remained on buprenorphine treatment at 8 weeks postrelease, an increase in treatment exposure that had higher rates of opioid-negative urine samples. Daily SLB is a familiar, effective, and evidence-based OUD therapy known to reduce heroin use and likely decrease the risk of overdose and death following release from jail or prison. These same treatment effects appear to translate to the newer XRB formulation per this pilot trial in a US correctional setting.

This study is in line with other previous randomized trials and cohort evaluations of methadone, buprenorphine, and naltrexone therapies for soon-to-be-released opioid dependent persons in prisons and jails, all of which have demonstrated feasibility and a clear advantage to prerelease medication initiation.^6,8,14,17,18,19,20^ OUD has 3 effective FDA-approved medications and several buprenorphine formulations. It is critical that MOUD uptake and access in US corrections expands. We are further evaluating XRB vs XR-Naltrexone in a multisite OUD treatment trial among adults involved in the criminal justice system.^21^

A stark difference between XRB and SLB was the mean number of jail medical clinic visits required per day, each visit requiring both medical and correctional staff. After induction, the XRB arm was in clinic about once every 10 days, compared with more than once per day for SLB. SLB participants also needed regular clinical evaluations for dose adjustments, whereas XRB has only 1 recommended starting dose. In a controlled environment concerned with buprenorphine diversion, these were clear advantages to XRB.

However, the acceptability and comparative effectiveness results from this pilot study may not translate directly into real world outcomes, or to correctional systems less experienced with opioid agonist treatments. Following the end of study recruitment in December 2019, CHS began to offer routine XRB as standard MOUD treatment. Administration of XRB was streamlined by training nursing staff so they could meet with eligible patients, counsel them, and offer injections at routine follow-up visits. Although OTP nurses were reassigned to assist with various needs during the COVID-19 pandemic response and were not meeting with eligible patients on a routine basis for at least 7 months, XRB continued to be offered through the OTP counselling staff who would then have a medical professional meet with the patient to offer XRB. Aftercare referrals were to community clinics providing SLB treatment; continued community XRB was not guaranteed. In these conditions, few individuals chose XRB, with the vast majority preferring daily SLB. The preference of SLB over XRB was likely because of a number of reasons, including the pause in routine follow-up visits for patients due to reassignment of nursing staff, novelty of the drug formulation, apprehension toward administration by subcutaneous injection, and patients’ desire to have a daily, oral medication as a way for treating their OUD as opposed to a monthly injection.

Some of the pharmacologic and logistical benefits of XRB were clear in this pilot: XRB reduces the risk of medication diversion and need for observed medication administration in correctional settings when compared with SLB. However, based on CHS’s experience offering both XRB and SLB, we recommend XRB availability in addition to SLB in correctional settings. This provides patients with multiple MOUD options, meets individuals where they are at, and may maximize MOUD uptake. XRB’s label requires patients to be tolerant of 8 mg/d or higher of SLB, and we recruited only adults already on a stable regimen of SLB for this pilot, further demonstrating a practical need for both formulations. While XRB offers a range of benefits and new features, it will not be the right choice for all incarcerated OUD patients. Among several good options, the best treatment is probably the one the patient prefers.

### Limitations

This proof-of-concept study had several limitations that are typical of open-label and unmasked pilots, including a small sample size, single jail and community sites, and study staff who provided care while also gathering data. Postrelease XRB was only available at the Bellevue treatment center, whereas some SLB participants followed up with providers throughout NYC; this likely dictated more missed research visits and missing urine tests in the SLB arm. However, community pharmacy buprenorphine prescription data for community treatment retention was universally available. Larger data sets and more reporting from correctional demonstration projects are needed to confirm and explore these initial findings of XRB’s acceptability and comparable effectiveness. Retention outcomes beyond 8 weeks are clearly important, as is the study of XRB’s wider dissemination and any potential cost-effectiveness.

## Conclusions

In this initial pilot proof-of-concept comparative effectiveness study, XRB demonstrated several potential advantages compared with standard daily sublingual buprenorphine, notably fewer clinic and medication administration visits during the remaining jail incarceration, and possibly an improved and longer-acting bridge of medication adherence and treatment retention during the weeks immediately following release from jail.

## Notes

Supplement 1.
Trial Protocol

Supplement 2.
Data Sharing Statement

## References

[1] Mattson CL, Tanz LJ, Quinn K, Kariisa M, Patel P, Davis NL. Trends and geographic patterns in drug and synthetic opioid overdose deaths—United States, 2013-2019. MMWR Morb Mortal Wkly Rep. 2021;70(6):202-207. doi:10.15585/mmwr.mm7006a4 33571180 PMC7877587

[2] National Center for Disease Statistics. Vital Statistics Rapid Release—Provisional Drug Overdose Death Counts. Centers for Disease Control and Prevention. Last updated July 14, 2021. Accessed May 27, 2021. https://www.cdc.gov/nchs/nvss/vsrr/drug-overdose-data.htm

[3] Winkelman TNA, Chang VW, Binswanger IA. Health, polysubstance use, and criminal justice involvement among adults with varying levels of opioid use. JAMA Netw Open. 2018;1(3):e180558. doi:10.1001/jamanetworkopen.2018.0558 30646016 PMC6324297

[4] Merrall EL, Kariminia A, Binswanger IA, . Meta-analysis of drug-related deaths soon after release from prison. Addiction. 2010;105(9):1545-1554. doi:10.1111/j.1360-0443.2010.02990.x 20579009 PMC2955973

[5] Binswanger IA, Blatchford PJ, Mueller SR, Stern MF. Mortality after prison release: opioid overdose and other causes of death, risk factors, and time trends from 1999 to 2009. Ann Intern Med. 2013;159(9):592-600. doi:10.7326/0003-4819-159-9-201311050-00005 24189594 PMC5242316

[6] Alex B, Weiss DB, Kaba F, . Death after jail release. J Correct Health Care. 2017;23(1):83-87. doi:10.1177/1078345816685311 28040993

[7] Kinlock TW, Gordon MS, Schwartz RP, Fitzgerald TT, O’Grady KE. A randomized clinical trial of methadone maintenance for prisoners: results at 12 months postrelease. J Subst Abuse Treat. 2009;37(3):277-285. doi:10.1016/j.jsat.2009.03.002 19339140 PMC2803487

[8] Moore KE, Roberts W, Reid HH, Smith KMZ, Oberleitner LMS, McKee SA. Effectiveness of medication assisted treatment for opioid use in prison and jail settings: a meta-analysis and systematic review. J Subst Abuse Treat. 2019;99:32-43. doi:10.1016/j.jsat.2018.12.003 30797392 PMC6391743

[9] Lee JD, Grossman E, Truncali A, . Buprenorphine-naloxone maintenance following release from jail. Subst Abus. 2012;33(1):40-47. doi:10.1080/08897077.2011.620475 22263712 PMC3310898

[10] Nunn A, Zaller N, Dickman S, Trimbur C, Nijhawan A, Rich JD. Methadone and buprenorphine prescribing and referral practices in US prison systems: results from a nationwide survey. Drug Alcohol Depend. 2009;105(1-2):83-88. doi:10.1016/j.drugalcdep.2009.06.015 19625142 PMC2743749

[11] Lee JD, Rich JD. Opioid pharmacotherapy in criminal justice settings: now is the time. Subst Abus. 2012;33(1):1-4. doi:10.1080/08897077.2011.616797 22263707

[12] Tomasino V, Swanson AJ, Nolan J, Shuman HI. The Key Extended Entry Program (KEEP): a methadone treatment program for opiate-dependent inmates. Mt Sinai J Med. 2001;68(1):14-20. 11135501

[13] Harris A, Selling D, Luther C, . Rate of community methadone treatment reporting at jail reentry following a methadone increased dose quality improvement effort. Subst Abus. 2012;33(1):70-75. doi:10.1080/08897077.2011.620479 22263715

[14] Magura S, Lee JD, Hershberger J, . Buprenorphine and methadone maintenance in jail and post-release: a randomized clinical trial. Drug Alcohol Depend. 2009;99(1-3):222-230. doi:10.1016/j.drugalcdep.2008.08.006 18930603 PMC2658719

[15] US Food and Drug Administration. FDA Approves SUBLOCADETM (Buprenorphine Extended-Release), the First and Only Once-Monthly Injectable Buprenorphine Formulation to Treat Moderate to Severe Opioid Use Disorder. Released November 30, 2017. Accessed August 6, 2021. https://www.fda.gov/news-events/press-announcements/fda-approves-first-once-monthly-buprenorphine-injection-medication-assisted-treatment-option-opioid

[16] Haight BR, Learned SM, Laffont CM, ; RB-US-13-0001 Study Investigators. Efficacy and safety of a monthly buprenorphine depot injection for opioid use disorder: a multicentre, randomised, double-blind, placebo-controlled, phase 3 trial. Lancet. 2019;393(10173):778-790. doi:10.1016/S0140-6736(18)32259-1 30792007

[17] Hedrich D, Alves P, Farrell M, Stöver H, Møller L, Mayet S. The effectiveness of opioid maintenance treatment in prison settings: a systematic review. Addiction. 2012;107(3):501-517. doi:10.1111/j.1360-0443.2011.03676.x 21955033

[18] McKenzie M, Zaller N, Dickman SL, . A randomized trial of methadone initiation prior to release from incarceration. Subst Abus. 2012;33(1):19-29. doi:10.1080/08897077.2011.609446 22263710 PMC3278074

[19] Springer SA, Qiu J, Saber-Tehrani AS, Altice FL. Retention on buprenorphine is associated with high levels of maximal viral suppression among HIV-infected opioid dependent released prisoners. PLoS One. 2012;7(5):e38335. doi:10.1371/journal.pone.0038335 22719814 PMC3365007

[20] Lee JD, McDonald R, Grossman E, . Opioid treatment at release from jail using extended-release naltrexone: a pilot proof-of-concept randomized effectiveness trial. Addiction. 2015;110(6):1008-1014. doi:10.1111/add.12894 25703440

[21] Waddell EN, Springer SA, Marsch LA, . Long-acting buprenorphine vs. naltrexone opioid treatments in CJS-involved adults (EXIT-CJS). J Subst Abuse Treat. Published online April 8, 2021. doi:10.1016/j.jsat.2021.108389 33865691 PMC8384640

