# Supplementary material for: Comparison of Treatment Retention of Adults With Opioid Addiction Managed With Extended-Release Buprenorphine vs Daily Sublingual Buprenorphine-Naloxone at Time of Release From Jail
Source: JAMA Netw Open. 2021 Sep 8;4(9):e2123032. doi: 10.1001/jamanetworkopen.2021.23032 (PMC8427378; doi:10.1001/jamanetworkopen.2021.23032)
Supplement: Supplement 1. — Trial Protocol [file jamanetwopen-e2123032-s001.pdf]

Lee JD, Malone M, McDonald RD, et al. Comparison of treatment retention of adults with opioid addiction managed with extended-release buprenorphine vs daily sublingual buprenorphine-naloxone at time of release from jail. *JAMA Network Open*. 2021;4(9):e2123032. doi:10.1001/jamanetworkopen.2021.23032

CLINICAL STUDY PROTOCOL

Buprenorphine extended-release in jail and at re-entry: pilot proof-of-concept open-label randomized controlled trial vs. daily sublingual buprenorphine-naloxone

Clinical Phase

Phase IV

Sponsor

National Institute on Drug Abuse

Protocol Version

December 11, 2019

version 10

Confidentiality Statement:

This study will be conducted in accordance with the Code of Federal Regulations on the Protection of Human Subjects (45 CFR Part 46), any other applicable US government research regulations, and institutional research policies and procedures. The Principal Investigator will assure that no deviation from, or changes to the protocol will take place without prior agreement from the sponsor and documented approval from the Institutional Review Board (IRB), except where necessary to eliminate an immediate hazard(s) to the trial participants. All personnel involved in the conduct of this study have completed Human Subjects Protection Training.

Study Personnel

Principal Investigator

Joshua D Lee
New York University
Email: Joshua.Lee@nyulangone.org

Co-Investigators

Jonathan Giftos, MD
NYC Health+Hospitals
Email: giftosj@nychhc.org

Ross MacDonald, MD
NYC Health+Hospitals
Email: rmacdonald@nychhc.org

Key Study Personnel:

Ryan Mcdonald
New York University
Email: Ryan.Mcdonald@nyulangone.org

Monica Malone
New York University
Email: Mia.Malone@nyulangone.org

Nadina Santana-Correa
New York University
Email: Nadina.Santana-Correa@nyulangone.org

Wanda Bonilla
New York University
Email: Wanda.Bonilla@nyulangone.org

Synopsis

Primary Objective

Feasibility and Implementation: Estimate the feasibility and ease of use of XRB vs. SLB as measured by initial patient preference and acceptability, in-treatment patient satisfaction, patient qualitative interviews, jail visit frequency and program cost estimates, and rates of medication diversion.

Secondary Objectives

Treatment Retention: Estimate the effectiveness of XRB vs. SLB in improving rates of community treatment initiation/continuation post-release.

Clinical Effectiveness: Estimate the effectiveness of XRB vs. SLB in decreasing illicit opioid (e.g. heroin) use post-release.

Naturalistic Comparative Effectiveness: Evaluate outcomes of XRB vs. SLB in the context of the XRNTX, ETAU, and Methadone outcomes accrued in the larger SOMATICS-XOR RCT.

Primary Outcome Variables

1. Initial patient preference and acceptability measured via baseline, initial patient interviews

2. In-treatment patient satisfaction measured using follow-up surveys, qualitative interviewing, and clinical research forms

3. Jail visit frequency and program cost estimates

4. Rates of medication diversion.

Secondary and Exploratory Outcome Variables

1. XRB vs. SLB in improving rates of community treatment initiation/continuation post-release.

2. Estimate the effectiveness of XRB vs. SLB in decreasing illicit opioid (e.g. heroin) use post-release.

3. Evaluate outcomes of XRB vs. SLB in the context of the XRNTX, ETAU, and Methadone outcomes accrued in the larger SOMATICS-XOR RCT

Study Duration

June 28, 2019 — June 27, 2020

Study Design

This Administrative Supplement proposal takes advantage of the existing SOMATICS-XOR U01 (NIDA) trial design and infrastructure to conduct a new pilot, N=50, proof-of-concept, open-label, non-blinded randomized controlled trial of XRB vs. SLB.

Study Population

Adult jail inmates with upcoming release date, current opioid dependence and currently maintained on sublingual buprenorphine-naloxone. N=50.

Number of Participants

N=50

Study Flowchart

Prior to Enrollment
Visit 0
Screening

Total N: 50

Obtain informed consent and HIPAA Authorization. Screen potential subjects by inclusion and exclusion criteria; obtain medical history, document informed consent and confirmation of eligibility in source records.

Visit 0
Baseline

Perform Baseline Assessments

ASILite, Timeline Followback, Demographics, MOUD Preference Form, Electronic Medical Records medical and psychiatric history

Randomization

Buprenorphine Extended-Release

Sublingual Buprenorphine

Visit 1-4
Follow-Up Treatment

Follow-Up Treatment Visits

Administrative data for SLB medication refills and post-release XRB injections, Timeline Followback, Urine specimen collection & testing, adverse event and serious adverse even reporting, NYS prescription monitoring data for community SLB refill reporting, open ended-interview, treatment retention. *At Visit 3, week 4 second XRB administered*

Visit 5
Final Study Visit

Final Study Visit

Medication is not dispensed. Timeline Followback, treatment retention, Urine specimen collection & testing, adverse event and serious adverse event reporting, NYS prescription monitoring data for community SLB refill reporting, open- ended interviews with participants concerning XRB vs. SLB treatment satisfaction and ease-of-use

Visit 6-10
Extension Study Treatment Visits

XRB Extension Treatment Visits

Administrative data for initial and/or additional XRB injections, Timeline Followback, Urine specimen collection & testing, adverse event and serious adverse even reporting. *At Visit 7 and 10, pregnancy test, qualitative interviews for eligible XRB participants*

Visit 11
Extension Study Final Visit

XRB Extension Final Safety Visit

Adverse event and serious adverse even reporting. Timeline Followback, Treatment Satisfaction, Qualitative Interviews if applicable.

Table of Contents

1 - Introduction 9
1.1 Introductory Statement 9
2 - Background 10
2.1.1 Preclinical Experience 10
2.1.2 Clinical Experience 10
2.2 Background/prevalence of research topic 10
3 - Rationale/Significance 11
3.1 Problem Statement 11
3.2 Purpose of Study/Potential Impact 11
3.3.1 Potential Benefits 13
3.3.2 Potential Risks 14
4 - Study Objectives 15
4.1 Hypothesis 15
4.2 Primary Objective 15
4.3 Secondary Objectives 15
5 - Study Design 16
5.1 General Design 16
5.1.1 Study Duration 16
5.1.2 Number of Study Sites 16
5.2.1 Primary Outcome Variables 16
5.2.2 Secondary and Exploratory Outcome Variables 17
5.3 Study Population 17
5.3.1 Number of Participants 17
5.3.2 Eligibility Criteria/Vulnerable Populations 17
6 - Methods 19
6.1.1 Identity of Investigational Product/New Drug 19
6.1.2 Dosage, Admin, Schedule 19
6.1.3 Method of Assignment/Randomization 19
6.1.4 Blinding and Procedures for Unblinding 19
6.1.5 Packaging/Labelling 20

6.1.6 Storage Conditions 20
6.1.7 Concomitant therapy 20
6.1.8 Restrictions 20
6.2 Assessments 21
6.2.1 Efficacy 21
6.2.2 Safety/Pregnancy-related policy 21
6.2.2.1 Adverse Events Definition and Reporting 21
6.2.3 Pharmacokinetics 23
6.2.4 Biomarkers 23
6.3 Study Procedures 23
6.3.1 Study Schedule 23
6.3.2 Informed Consent 27
6.3.3 Screening 27
6.3.4 Recruitment, Enrollment and Retention 27
6.3.5 On Study Visits 28
6.3.6 End of Study and Follow-up 30
6.3.7 Removal of subjects 30
6.4.1 Statistical Design 30
6.4.2 Sample Size Considerations 31
6.4.3 Handling of Missing Data 31
7 - Trial Administration 32
7.1 Ethical Considerations 32
7.2 Institutional Review Board (IRB) Review 32
7.3 Subject Confidentiality 32
7.4 Deviations/Unanticipated Problems 33
7.5 Data Quality Assurance 34
7.5.1 Data Collection 34
7.5.1.1 Access to Source 34
7.5.1.2 Data Storage/Security 36
7.6 Study Records 36
7.6.1 Retention of Records 36
7.7 Study Monitoring 37

7.8 Data Safety Monitoring Plan 37
7.9 Study Modification 37
7.10 Study Discontinuation 37
7.11 Study Completion 38
7.12 Conflict of Interest Policy 38
7.13 Funding Source 38
Appendices 39
List of Tables 40
References 41

1 - Introduction

1.1 Introductory Statement

This is a pilot proof-of-concept randomized controlled trial, open-label and unblinded, examining the feasibility and acceptability of Buprenorphine extended-release vs. daily sublingual buprenorphine-naloxone for the treatment of opioid use disorder in jail and at community re-entry.

2 - Background

2.1.1 Preclinical Experience

Buprenorphine, a partial μ-opioid receptor agonist, was approved by the Food and Drug Administration in 2002 as office-based pharmacotherapy for opioid dependence. As an opioid partial agonist, buprenorphine can produce some opioid-like effects such as euphoria or respiratory depression, however, its maximal effects are less than those of full agonists like heroin and methadone. At low doses Buprenorphine produces sufficient agonist effect to enable opioid-addicted individuals to discontinue the misuse of opioids without experiencing withdrawal symptoms. Buprenorphine's opioid effects increase with each dose until at moderate doses they level off, even with further dose increases. This "ceiling effect" lowers the risk of misuse, dependency, and side effects (1).

2.1.2 Clinical Experience

Monthly, injectable, buprenorphine extended-release (XRB, Sublocade™, Indivior) is the most recently approved pharmacotherapy for opioid use disorders in the U.S.(2). XRB is available as of March-2018 in a pre-mixed subcutaneous formulation, administered monthly, and equivalent to a 16-24mg/day maintenance dose of sublingual buprenorphine-naloxone (SLB). In this pilot proposal, Buprenorphine extended-release (XRB) or sublingual buprenorphine (SLB) daily maintenance will be provided per usual Opioid Treatment Program protocols in-jail and prior to release. Both medications will be available through the study and the Bellevue Hospital Addiction Medicine clinic free-of-charge. XRB consists of a once monthly abdominal subcutaneous injection using a pre-filled syringe, per usual package insert instructions. SLB is a delivered daily by observed dosing in-jail (controlled substances are not self-administered in-jail). Post-release, all participants may elect to continue SLB maintenance with the Bellevue Primary Care Addiction Medicine clinic, or may pursue SLB maintenance from non-NYU/Bellevue community providers. SLB is available free of charge to uninsured patients at Bellevue Hospital Center, or participants may fill SLB prescriptions at community pharmacies. SLB medication will not be provided by the study itself.

2.2 Background/prevalence of research topic

In 2016, opioids were involved in 42,249 deaths and opioid overdose deaths were five times higher than in 1999 (3). On average, 115 Americans die every day from an opioid overdose (4). The opioid and overdose epidemic has intensified efforts to expand and optimize effective medication treatment for opioid use disorder (methadone, extended-release naltrexone, buprenorphine) in criminal justice and primary care populations. Despite the effectiveness of methadone and buprenorphine maintenance as standard of care in NYC jails, only about 30% of individuals initiating buprenorphine maintenance in-jail successfully link to community treatment post-release. XRB is newly FDA approved and its effectiveness in a criminal justice setting is promising but untested.

3 - Rationale/Significance

3.1 Problem Statement

The opioid epidemic in the United States continues to worsen. New York City currently has a robust methadone and buprenorphine maintenance program for adults with opioid use disorder (OUD). However, despite these standards-of-care, outcomes for all OUD patients, including buprenorphine patients, in NYC following release from jail are in need of improvement. Overdose rates in NYC continue to worsen, including among recently incarcerated individuals (5). Only about 30% of individuals initiating buprenorphine maintenance for opioid use disorders (OUDs) in-jail currently successfully link to community treatment post-release (6), which has been a finding in a previous NIDA clinical trial and observational studies we have conducted at NYU-Bellevue (7, 8).

3.2 Purpose of Study/Potential Impact

Monthly, injectable, buprenorphine extended-release (XRB, Sublocade™, Indivior) is the most recently approved pharmacotherapy for opioid use disorders in the US. XRB is available as of March-2018 in a pre-mixed subcutaneous formulation, administered monthly, and equivalent to a 16-24mg/day maintenance dose of sublingual buprenorphine-naloxone (SLB).

XRB offers several advantages to other forms of 'medication treatment for opioid use disorders' (MOUD), which currently consist of daily sublingual buprenorphine (Suboxone films, Zubsolv, or generic buprenorphine-naloxone tablets), daily oral methadone maintenance delivered in Opioid Treatment Programs, or extended-release naltrexone (XR-NTX). None of these earlier medications have proven to be 'killer apps', and uptake of MOUD in US correctional facilities (jails, prisons) and community-supervised populations (drug courts, parole programs) lag the public health need and high overdose risk in these settings and populations. Buprenorphine products in particular have been relatively shunned in CJS, likely due to staffing constraints required by observed induction and daily dosing, concerns regarding diversion and misuse, and common stigmas related to opioid agonist (methadone or buprenorphine) maintenance approaches (9).

NYC jails are an exception to this sub-par US CJS standard, in part due to the current leadership of this proposal's Co-Investigators at NYC Health+Hospitals Correctional Health Services, Ross MacDonald MD and Jonathan Giftos MD, who are, respectively, Chief Medical Officer and Clinical Director of Substance Use Treatment/Medical Director of the Opioid Treatment Program for all of NYC jails. NYC has a robust methadone and buprenorphine maintenance program for adults with OUD. Despite these standards-of-care, outcomes for all OUD patients, including buprenorphine patients, in NYC following release from jail are in need of improvement. Overdose rates in NYC continue to worsen, including among recently incarcerated individuals (5). Only about 30% of individuals initiating buprenorphine maintenance for opioid use disorders (OUDs) in-jail currently successfully link to community treatment post-release (6) which has been a finding in a previous NIDA clinical trial and observational studies we have conducted at NYU-Bellevue (7, 8) . Major obstacles to office-based opioid treatment (OBOT) with MOUD following re-entry include limited mobility and communication due to incarceration, administrative support for bridging

to a new clinic, high risks of drug/alcohol relapse, and rigid, impersonal treatment 'systems' which are often not customized to or welcoming of the recently incarcerated.

The new buprenorphine extended-release formulation (XRB) has several potential feasibility and effectiveness advantages vs. daily sublingual buprenorphine (SLB), including: 1) fewer overall medical, nursing, and pharmacy visits, fewer correctional staff hours, and potentially lower program costs vs. daily observed dosing, 2) near zero probability of diversion/misuse vs. frequent diversion, 3) an improved, long-acting 'bridge' of medication adherence at release, which is crucial as patients struggle to continue daily SLB adherence, avoid relapse, and connect to community treatment. The current NYC jail high-volume buprenorphine maintenance program provides an ideal setting in which to pilot XRB vs. SLB.

Rikers Island and the New York City Department of Corrections administered jail system in NYC is a prominent exception to US norms. NYC Health+Hospitals Division of Correctional Health Services, which administers all jail-based health care in NYC, and has a high-volume program for both continuing existing SLB patients through incarceration and initiating SLB in new patients prior to release. NYC jails provide an ideal setting in which to pilot jail-based use of newer extended-release buprenorphine compounds. Two members of NYC Health+Hospitals leadership, are key co-investigators in this proposal. This pilot will be a first of its kind evaluation of XRB in a criminal justice, underserved population at high risk or relapse and overdose.

3.3.1 Potential Benefits

This proposal will likely be the first or among the first high-quality RCT evaluations of XRB in a criminal justice setting. Both XRB and SLB standard of care are safe, efficacious medications for the treatment of OUDs. The expected benefits in the XRB group are fewer medication and nursing visits during incarceration, continuous long-acting medication treatment at release and prior to the first community treatment visit, and potentially a lower risk of relapse to heroin and other drug/alcohol use vs. SLB. In addition the XRB is delivered free of charge. The SLB treatment-as-usual group will receive the same compensation and encouragement to continue care both in custody and following release, which will be above and beyond usual care. Both arms, however, may experience no benefit from study participation, if the medications are not effective, acceptable, or if post-release follow-up is not pursued or completed. The extra XRB medical treatment, pre- and post-release counseling (both arms), and monetary compensation (both arms) is intended and unlikely to be coercive – that is, motivate persons to participate in the trial against their preferences or wishes. These benefits are on par with other recent and approved NYC jail opioid treatment trials which attempt to provide adequate but non-coercive benefits to all participants. The XRB medication free of charge or the modest monetary compensation is unlikely to sway an individual with other good options including jail-based methadone and buprenorphine treatment and no other interest in participating in or following up during the trial.

3.3.2 Potential Risks

Any relapse to illicit opioid and other drug and alcohol use among any of the two treatment arms after release from jail (or while incarcerated) implies a risk of death and disability, and some proportion of participants in all three arms can be expected not to respond to treatment and resume opioid and/or other drug and alcohol use. All participants relapsing post-release and struggling with on-going drug and alcohol use will be counseled and referred by staff to any appropriate treatment modalities, including the immediately available addiction services available at Bellevue Hospital (emergency detoxification with referral to residential treatment or supportive housing, methadone treatment, buprenorphine, intensive outpatient, dual diagnosis).

Sublingual Buprenorphine-Naloxone Treatment Arm. The risks of SLB are the usual risks experienced by any patient as part of standard of care buprenorphine maintenance, which is first-line treatment for opioid use disorders in NYC jails and in the community. Persons receiving SLB may experience several common side effects such as drowsiness, dizziness, constipation, or headaches. The SLB arm will otherwise not be exposed to risks beyond those of usual care, which will be provided by existing jail and community partners. Further, participants in the SLB arm will have already agreed to these risks and benefits of an SLB treatment program prior to recruitment and enrollment, as their choice to engage in jail SLB will have been established well before enrollment, which will occur at the end of the incarceration period.

Buprenorphine Extended-Release Treatment Arm. XRB carries the same potential systemic buprenorphine-related side effects as SRB. XRB also carries risks from the injection. Injection site soreness is possible and usually well tolerated. More severe injection site reactions, although rare, include swelling, itching, and pain. All injections sites will be inspected and monitored by study clinicians.

Concomitant use of buprenorphine and benzodiazepine or other CNS depressants increases the risk of adverse reactions including overdose, respiratory depression, and death. Urine toxicology biometric tests will be used to ensure persons receiving both SLB and XRB have not taken any other substances. Participants will be educated regarding the risks of concomitant use of benzodiazepines, sedatives, opioid analgesics and alcohol.

Additionally, if a participant requires opioids for any medical treatment once on buprenorphine, they will likely not have adequate pain relief from usual doses of opioid pain medications. Participants should tell the treating clinician that they are participating in this clinical study and will need non-opioid pain relief or higher doses of opioid pain medications for their condition, which they should receive only in a monitored medical setting, such as an emergency room. As a result, participants may experience higher level of pain.

Risk/Benefit Ratio: Most of the risks described are expected adverse events associated with XRB and SLB, or those of baseline opioid dependence and jail-to-community ETAU or MTP. The risks of the active treatment arms, XRB and SLB are likely small compared to the expected benefit of discontinuing opioid use.

4 - Study Objectives

4.1 Hypothesis

Primary Aim hypothesis: We hypothesize that XRB will be a feasible, effective, and preferred/accepted medication assisted therapy for OUDs in jail as compared to SLB.

Secondary Aim 1 hypothesis: We hypothesize that XRB will be more effective in improving rates of community treatment initiation/continuation post-release as compared to SLB.

Secondary Aim 2 Hypothesis: We hypothesize that XRB will be more effective in decreasing illicit opioid use post-release as compared to SLB.

Secondary Aim 3 Hypothesis: We hypothesize that the use of XRB vs. SLB will lead to better outcomes in the context of XRNTX, ETAU, and Methadone outcomes accrued in the larger SOMATICS-XOR RCT.

4.2 Primary Objective

Feasibility and Implementation: Estimate the feasibility and ease of use of XRB vs. SLB as measured by initial patient preference and acceptability, in-treatment patient satisfaction, patient qualitative interviews, jail visit frequency and program cost estimates, and rates of medication diversion.

4.3 Secondary Objectives

Treatment Retention: Estimate the effectiveness of XRB vs. SLB in improving rates of community treatment initiation/continuation post-release.

Clinical Effectiveness: Estimate the effectiveness of XRB vs. SLB in decreasing illicit opioid (e.g. heroin) use post-release.

Naturalistic Comparative Effectiveness: Evaluate outcomes of XRB vs. SLB in the context of the XRNTX, ETAU, and Methadone outcomes accrued in the larger SOMATICS-XOR RCT.

5 - Study Design

5.1 General Design

This is a phase IV, 8 week, pilot proof-of-concept randomized controlled trial, open-label and unblinded of XRB vs SLB (N=50) that takes advantage of the existing SOMATICS-XOR U01 (NIDA) trial design.

5.1.1 Study Duration

This is an 8 week pilot RCT.

The study has received 50 doses of in-kind Sublocade™ study medication from the manufacturer, Indivior as of September 13, 2019. With this new stock of medication, we would like to offer all participants the option of a therapeutic treatment extension of 24 weeks post study visit 5 week 8. Active treatment will continue after the Week 8 study visit through Week 20 wherein participants will be eligible to receive initial and/or additional XRB injections. A safety visit, in which no medication is administered, will be conducted at Week 24 to document safety reporting and provide post-study treatment referrals.

This 24 week therapeutic extension will be offered to all interested participants regardless of their treatment assignment after the completion of Visit 5 Week 8. Only Sublocade™ injections will be offered, no additional forms of compensation will be provided.

5.1.2 Number of Study Sites

This is a multi-site pilot, RCT. Participants (N=50) will be recruited from Rikers Island NYC Jail and will then complete all follow-up visits at Bellevue Hospital Center in NYC. The NYU School of Medicine Institutional Review Board (IRB) is serving as the IRB of record for Health+Hospitals involvement in this research study.

Sites: The NYC jail system houses approximately 8,000-9,000 inmates daily in 11+ inmate facilities, 8 of which are located on Rikers Island. Most (90%) are adults detained on charges or sentenced to misdemeanor charges and likely to leave jail and return to the community following an average pretrial detention of several weeks or after serving a misdemeanor sentence of less than one year. The jail Opioid Treatment Program primarily operates at three facilities, a male admission facility (Anna M Kross Center), a male sentenced-inmate facility (Eric M Taylor Center), and a single female facility (Rose M Singer Center), all located on Rikers Island in the Bronx. For the purposes of this pilot, participants will be recruited from the Eric M Taylor Center and the Rose M Singer Center. The jail Opioid Treatment Program currently treats around 100-200 patients daily with sublingual buprenorphine, this pilots target population, and 500-800 daily with methadone maintenance. All follow-up will be conducted at the Bellevue Hospital Center's Adult Primary Care Clinic and its Addiction Medicine clinic, which is currently a routine aftercare referral for jail patients continuing buprenorphine in the community and the site of the current NYU-NIDA U01 XOR trial. Patient’s lost-to-follow-up and out-of-contact are tracked by study staff in the community whenever possible, including in-person door-knocking by Study Staff throughout the 5 boroughs and northern New Jersey.

5.2.1 Primary Outcome Variables

The primary outcome of in-jail feasibility and acceptability will be measured by the percent of eligible participants who enroll in the study, the mean medical/medication visits per arm and

per participant, rates of medication diversion, and open-ended interviews with patients regarding treatment satisfaction and ease of use. Additional qualitative interviews will be conducted with eligible participants to further examine opioid use disorder treatment with extended-release buprenorphine, in-jail and within the community.

5.2.2 Secondary and Exploratory Outcome Variables

Secondary Aims 1-3 will be measured utilizing the following assessments: baseline demographics, baseline ASI-Lite, baseline MOUD (medication treatment for opioid use disorder) exposure/preference, Timeline Followback, urine toxicology results, treatment retention, and NYS Prescription Monitoring Data. XRNTX, ETAU, and Methadone outcomes will be taken from the SOMATICS-XOR U01 parent study.

5.3 Study Population

Adult jail inmates with upcoming release date, current opioid dependence and currently maintained on sublingual buprenorphine-naloxone (N=50).

5.3.1 Number of Participants

N=50. A total sample size of N=50 would allow for an estimate of an Odds Ratio of 2.0-3.0 for the rate of persons successfully in community buprenorphine treatment at 4 weeks post-release (71% vs. 30% success favoring XRB; two-sided alpha of 0.05; 80+% power).

5.3.2 Eligibility Criteria/Vulnerable Populations

<u>Inclusion criteria</u>

1) Adults ≥18yo incarcerated in NYC jails with known release dates.

2) DSM-V criteria for current opioid use disorder (DSM-IV opioid dependence).

3) Currently maintained on sublingual buprenorphine-naloxone in the NYC jail opioid treatment program.

<u>Exclusion Criteria</u>

1) Individual not interested in XRB treatment. Current SLB patients are otherwise by definition appropriate for XRB.

2) Pregnant or planning conception. As female participants are incarcerated & active in the jail opiate treatment program at the time of baseline assessments and treatment induction, a pregnancy test is not required by the study staff. A urine dipstick pregnancy (hCG) test will be administered at week 4 visit 3. The test detects human chorionic gonadotropin (hCG) in urine with a sensitivity/specificity of: 25 mIU hCG/ml, >99%. Time to result is four minutes. If negative, a urine pregnancy test will be administered at the final week 8 visit 5 and final week 20 visit 8 (if applicable) to ensure that a participant is not pregnant.

3) No severe or acute medical or psychiatric disability preventing safe study participation or making follow-up unlikely.

<u>Federal Research among Prisoners</u>. Study participants are clearly considered prisoners at the time of enrollment. The NYU SOM IRB will contact DHS/OHRP to address this topic and for permission to enroll prisoners in a federally funded research trial. However, we believe this study carefully conforms to the Federal guidelines for research among prisoners (CFR 46.306) in the following manner:

1.) Research on practices, both innovative and accepted, which have the intent and reasonable probability of improving the health or well-being of the subject. In cases in which those studies require the assignment of prisoners in a manner consistent with protocols approved by the IRB to control groups which may not benefit from the research, the study may proceed only after the Secretary has consulted with appropriate experts, including experts in penology medicine and ethics, and published notice, in the Federal Register, of his intent to approve such research.

2) Any possible advantages accruing to the prisoner through his or her participation in the research, when compared to the general living conditions, medical care, quality of food, amenities and opportunity for earnings in the prison, are not of such a magnitude that his or her ability to weigh the risks of the research against the value of such advantages in the limited choice environment of the prison is impaired. Incentives and benefits from participation are not coercive and are fair value of participant's time and, following release, travel.

(3) The risks involved in the research are commensurate with risks that would be accepted by non-prisoner volunteers. This study's methods and interventions are consistent with good clinical practices in community settings.

(4) Procedures for the selection of subjects within the prison are fair to all prisoners and immune from arbitrary intervention by prison authorities or prisoners. Unless the principal investigator provides to the Board justification in writing for following some other procedures, control subjects must be selected randomly from the group of available prisoners who meet the characteristics needed for that particular research project. Jail authorities have no role in this study or in treatment assignment, which is random.

(5) The information is presented in language which is understandable to the subject population. The informed consent and consent quiz are intended to be understandable to adults in jail.

(6) Adequate assurance exists that parole boards will not take into account a prisoner's participation in the research in making decisions regarding parole, and each prisoner is clearly informed in advance that participation in the research will have no effect on his or her parole; There is no role for, relationship, or other interaction with parole or probation authorities and this study.

(7) Where the Board finds there may be a need for follow-up examination or care of participants after the end of their participation, adequate provision has been made for such examination or care, taking into account the varying lengths of individual prisoners' sentences, and for informing participants of this fact. The study primarily consists of community follow-up of adults released from NYC jails. Follow-up consists of research visits conducted weekly then bi-weekly for both medication arms post-release (week 1 post-release, week 2, week 4, week 6, week 8). After the completion of Visit 5 Week 8, all participants will be eligible for a 24 week therapeutic extension study wherein they are offered initial and/or additional XRB injections. These will occur monthly post study week 8: week 12, week 16, week 20, week 24, and week 28. A final safety visit, no medication administered, will be conducted at week 32 to document safety reporting and provide post-study treatment referrals.

6 - Methods

6.1.1 Identity of Investigational Product/New Drug

Monthly, injectable, buprenorphine extended-release (XRB, Sublocade™, Indivior) is the most recently FDA approved pharmacotherapy for opioid use disorders in the U.S.(2). XRB is available as of March-2018 in a pre-mixed subcutaneous formulation, administered monthly, and equivalent to a 16-24mg/day maintenance dose of sublingual buprenorphine-naloxone (SLB). Sublingual buprenorphine was approved by the FDA in 2002 and is delivered daily by placing the medication under the tongue for 5 to 10 minutes and letting it dissolve completely.

6.1.2 Dosage, Admin, Schedule

Buprenorphine Extended Release: XRB is a 300mg or 100mg pre-mixed subcutaneous injectable formulation to be administered monthly. XRB is for abdominal subcutaneous injection only. Participants in the XRB treatment arm will be given 1 or more XRB injections prior to release from jail and one or more at weeks 4, 8, 12, and 16 post-release, depending on their release date.

Sublingual Buprenorphine: SLB (SUBOXONE, Zubsolv, or generic tablets) is a daily sublingual film or tablet ranging from 8-24mg/day or equivalent (Zubsolv is dosed 5.7-17.1 mg/day). The film or tablet is placed under the tongue for 5 to 10 minutes until dissolved completely. Participants in the SLB treatment arm will be provided SLB daily by observed dosing in-jail (controlled substances are not self-administered in-jail) and encouraged to continue SLB treatment in weekly, bi-weekly, or monthly quantities for unobserved, daily, self-administration through week 5. Patients may obtain SLB care free-of-charge from the Bellevue Hospital Center Addiction Medicine clinic or from non-NYU/Bellevue providers and pharmacies per usual care standards. After the completion of Visit 5 Week 8, SLB participants will be offered XRB treatment in the 20 week therapeutic extension. If interested, participants would receive one or more XRB injections during the period of Weeks 8, 12, 16, 20, 24, and 28 followed by a final safety documentation visit at Week 32.

6.1.3 Method of Assignment/Randomization

Screening and Randomization

This pilot will recruit from within the standard-of-care NYC jail opioid treatment program, led by the program director, Jonathan Giftos, MD. Potential participants currently maintained on sublingual buprenorphine (currently a NYC jail standard of care) will be offered study information and encouraged to enroll. Interested participants will be referred by the Opioid Treatment Program staff to Study Staff for further education about the trial and to schedule written informed consent and a comprehensive screening visit. Consented and eligible participants will be randomized by sealed envelope or a web-based randomizer 1:1 to the 2 study cells: XRB and SLB.

6.1.4 Blinding and Procedures for Unblinding

This is an 8-week, open-label, single site, proof-of-concept randomized controlled trial. There is no blinding of treatment assignment or of outcomes assessments.

6.1.5 Packaging/Labeling

Buprenorphine Extended Release (SUBLOCADE): XRB is available in dosage strengths of 100 mg/0.5 mL and 300 mg/1.5 mL buprenorphine. Each dose is provided in a prefilled syringe with a 19 gauge 5/8-inch needle. The recommended dose of XRB following induction and dose adjustment with transmuscosal buprenorphine is 300 mg monthly for the first two months followed by a maintenance dose of 100 mg or 300mg monthly. We will offer both 300mg and 100mg doses pre-release and at week 4 post-release. Each dose is supplied in an individual kit and will be acquired in bulk shipments from the supplier Indivior.

At the outset of this study and original IRB approval, it was proposed that each participant randomized to the XRB (SUBLOCADE) treatment arm would receive up to two injections of XRB, each 300mg according to the medication package insert. However, the study has since been modified to include a therapeutic treatment extension of 24 additional weeks post Visit 5 Week 8 in which initial and/or additional XRB injections would be offered to all interested participants (see section 5.1.1). With this therapeutic extension, eligible & interested participants could receive 4+ XRB injections in the community. With this in mind, the study must expand the planned XRB dosage to include the 100mg formulation of XRB. As the XRB package insert states, the recommended dose of XRB following induction is 300mg monthly for the first two months followed by a maintenance dose of 100mg or 300mg monthly. The study must have the capability to offer eligible participants receiving a third XRB injection the lower, recommended maintenance dose of 100mg in addition to the standard 300mg.

Sublingual Buprenorphine (SUBOXONE, Zubsolv, or generic tablets): SLB is administered sublingually or buccally as a single daily dose. Medication should be prescribed in consideration of the frequency of visits. Provision of multiple refills is not advised in early treatment or without appropriate follow-up visits. After treatment induction and stabilization, the maintenance dose of SLB is generally in the range of 4mg/1mg buprenorphine/naloxone to 24mg/6mg buprenorphine/naloxone per day depending on the individual patient and clinical response, as determined by the clinician. Daily doses will vary by individual. The recommended target dose of SLB during maintenance is 16mg/4mg buprenorphine/naloxone. SLB will be prescribed and provided via observed dosing in-jail (controlled substances are not self-administered in-jail) per usual care protocols. Post-release, all participants may elect to continue SLB maintenance with the Bellevue Primary Care Addiction Medicine clinic, or may pursue SLB maintenance from non-NYU/Bellevue community providers. SLB is available free of charge to uninsured patients at Bellevue Hospital Center, or participants may fill SLB prescriptions at community pharmacies. SLB medication will not be provided by the study itself. Self-report, BHC EMR records, and NYS Prescription Monitoring Plan audits will document SLB post-release community treatment retention, daily dose, and refill frequencies.

6.1.6 Storage Conditions

XRB Storage Conditions: Store refrigerated at 2 - 8ºC (35.6 - 46.4ºF). Once outside the refrigerator XRB may be stored in its original package at room temperature 15-30ºC (59-86ºF), for up to 7 days prior to administration.

SLB Storage Conditions: Store at room temperature between 20 - 25ºC (68 - 77ºF).

6.1.7 Concomitant therapy

It is advisable for both XRB and SLB treatment arms that patients refrain from use of benzodiazepines, sedatives, tranquilizers, antidepressants, stimulants, or alcohol. Urine toxicology will be collected at medication dispensation visits in order to ensure no contraindications.

6.1.8 Restrictions

XRB Restrictions: For abdominal subcutaneous injection only. Do not administer XRB intravenously or intramuscularly. Only a healthcare provider should prepare and administer XRB. Administer monthly with a minimum of 26 days between doses.

SLB Restrictions: Take the prescribed dose once a day. SLB must be taken whole. Do not cut, chew, or swallow. SLB film should not be moved after placement. Proper administering technique should be demonstrated to the patient by a healthcare provider.

6.2 Assessments

Measures and Assessments:

a) Baseline assessments include a drug and alcohol use history (ASI-Lite), a pre-arrest recall of drug and alcohol use (Timeline Followback), a medication treatment for opioid use disorder exposure/preference form (MOUD preference), demographic information, and jail Electronic Medical Record medical and psychiatric history and laboratory data, including HIV and HCV status.

b) In-jail feasibility assessments of XRB vs. SLB include logging the % eligible enrolling in the study; the density of medical/nursing/pharmacy visits per arm and per participant; the incidence rates of medication diversion; open-ended interviews with participants surrounding XRB vs. SLB and ease-of-use and satisfaction;

a) Post-release follow-up assessments include administrative data for SLB medication refills and post-release XRB injections, self-reported opioid and other drug use (Timeline Followback), urine toxicology testing, pregnancy testing, treatment retention (treatment retention form), Adverse Event and Serious Adverse Event monitoring (SAFTEE), and NYS Prescription Monitoring Data for community SLB pharmacy refill reporting. The I-STOP/PMP (prescription monitoring program) registries will be accessed in order to track participant data related to respective treatments. Additional qualitative interviews will be conducted with eligible XRB participants to further examine opioid use disorder treatment with extended-release buprenorphine, in-jail and within the community.

6.2.1 Efficacy

The primary outcome of in-jail feasibility and acceptability will be measured by the percent of eligible participants who enroll in the study, the mean medical/medication visits per arm and per participant, the incidence rates of medication diversion, and open-ended interviews with patients regarding treatment satisfaction and ease of use.

Secondary Aims 1-3 will be measured utilizing the following assessments: baseline demographics, baseline ASI-Lite, baseline MOUD exposure/preference, Timeline Followback, urine toxicology results, treatment retention, and NYS Prescription Monitoring Data. XRNTX, ETAU, and Methadone outcomes will be taken from the SOMATICS-XOR U01 parent study.

6.2.2 Safety/Pregnancy-related policy

XRB and SLB are both FDA approved, safe, efficacious medications for the treatment of opioid use disorder. As with most medications both XRB and SLB carry side effects as detailed above. Participants will be monitored throughout this pilot RCT by study physicians, all adverse events and/or serious adverse events will be documented and medication will be discontinued if necessary. Women who are pregnant or plan to become pregnant will be excluded from this pilot RCT.

6.2.2.1 Adverse Events Definition and Reporting

For the purposes of this pilot, proof-of-concept, RCT, an adverse event (AE) is any symptom, sign, illness or experience that develops or worsens in severity during the course of the study. Intercurrent illnesses or injuries should be regarded as adverse events.

Abnormal results of diagnostic procedures are considered to be adverse events if the abnormality:

- results in study withdrawal
- is associated with a serious adverse event
- is associated with clinical signs or symptoms
- leads to additional treatment or to further diagnostic tests
- is considered by the investigator to be of clinical significance (i.e. medication side effects)

All adverse events will be documented in the attached clinical research form and reported to the IRB where necessary.

A ***serious adverse event*** (SAE) is any AE that is:

- fatal
- life-threatening
- requires or prolongs hospital stay
- results in persistent or significant disability or incapacity
- a congenital anomaly or birth defect
- an important medical event

Important medical events are those that may not be immediately life threatening, but are clearly of major clinical significance. They may jeopardize the subject, and may require intervention to prevent one of the other serious outcomes noted above. For example, drug overdose or abuse, a seizure that did not result in in-patient hospitalization, or intensive treatment of bronchospasm in an emergency department would typically be considered serious.

All serious adverse events will be documented in the attached clinical research form and reported to the IRB where necessary.

Classification of an Adverse Event: for AEs in this pilot RCT the following guidelines will be used to describe severity.

- Mild — Events require minimal or no treatment and do not interfere with the participant's daily activities.
- Moderate — Events result in a low level of inconvenience or concern with the therapeutic measures. Moderate events may cause some interference with functioning.
- Severe — Events interrupt a participant's usual daily activity and may require systemic drug therapy or other treatment. Severe events are usually potentially life-threatening or incapacitating.

Relationship to Study Medication: To assess whether an AE may be associated with the study medication (XRB vs. SLB) the following guidelines will be used:

- Definitely Related — There is clear evidence to suggest a causal relationship, and other possible contributing factors can be ruled out. The clinical event, including an abnormal laboratory test result, occurs in a plausible time relationship to drug administration and cannot be explained by concurrent disease or other drugs or chemicals. The response to withdrawal of the drug (dechallenge) should be clinically plausible. The event must be pharmacologically or phenomenologically definitive, with use of a satisfactory rechallenge procedure if necessary
- Probably Related — There is evidence to suggest a causal relationship, and the influence of other factors is unlikely. The clinical event, including an abnormal laboratory test result, occurs within a reasonable time after administration of the drug, is unlikely to be attributed to concurrent disease or other drugs or chemicals, and follows a clinically reasonable response on withdrawal (dechallenge). Rechallenge information is not required to fulfill this definition.
- Possibly Related — There is some evidence to suggest a causal relationship (e.g., the event occurred within a reasonable time after administration of the trial medication). However, other factors may have contributed to the event (e.g., the participant's clinical condition, other concomitant events). Although an AE may rate only as "possibly related" soon after discovery, it can be flagged as requiring more information and later be upgraded to "probably related" or "definitely related," as appropriate.
- Unlikely to be related — A clinical event, including an abnormal laboratory test result, whose temporal relationship to drug administration makes a causal relationship improbable (e.g., the event did not occur within a reasonable time after administration of the trial medication) and in which other drugs or chemicals or underlying disease provides plausible explanations (e.g., the participant's clinical condition, other concomitant treatments).
- Not Related — The AE is completely independent of study drug administration, and/or evidence exists that the event is definitely related to another etiology. There must be an alternative, definitive etiology documented by the clinician.

Study clinicians will be responsible for determining whether an AE is expected or unexpected. An AE will be considered unexpected if the nature, severity, or frequency of the event is not consistent with the risk information previously described for the study agent.

The pilot study team will record all reportable events with start dates occurring any time after informed consent is obtained until 7 (for non-serious AEs) or 30 days (for SAEs) after the last day of study participation. At each study visit, the investigator will inquire about the occurrence of AE/SAEs since the last visit. Events will be followed for outcome information until resolution or stabilization.

6.2.3 Pharmacokinetics

Not applicable.

6.2.4 Biomarkers

Not applicable.

6.3 Study Procedures

The study procedures are detailed below.

6.3.1 Study Schedule

The total number of expected visits per participant is 6 visits. All participants will be given the option to participate in a 24 week therapeutic extension study of XRB treatment following completion of Visit 5 Week 8. If a participant elects to continue and/or initiate XRB treatment, there will be 12 total visits per participant. The average time needed to complete each visit will be approximately one hour. The initial week 0 screening and randomization visit will be conducted in a Rikers Island jail facility in the Opioid Treatment Program. Once consented, enrolled, and randomized, buprenorphine extended-release (XRB) or sublingual buprenorphine (SLB) daily maintenance will provided per usual Opioid Treatment Program protocols in-jail and prior to release. The timing between randomization and release will vary. Typical misdemeanor sentences in NYC are 30-60 days, and participants may randomize at any time during this incarceration period, begin XRB vs. SLB treatment, and then leave jail after weeks-to-months. This variable, naturalistic, in-custody study period remains Study Week 0. Study Week 1 commences with the day of release from jail. Post-release, all study visits will be conducted at Bellevue Hospital Center's (BHC) Addiction Medicine clinic. Medical and research visits will be conducted weekly and then bi- weekly for both medication arms post-release (week 1 post-release, week 2, week 4, week 6, week 8). The medication schedule for both arms is as follows:

Buprenorphine Extended-Release: Participants randomized to this arm will receive at least two doses of XRB. The first will be administered at study week 0 in-jail, at least one week prior to release. Participants incarcerated for greater than 4 weeks following randomization will receive 2+ XRB doses prior to release. Post-release, XRB injections will be administered at study week 1-16 in the BHC Addiction Medicine clinic depending on the timing of the previous pre-release/community injections.

Sublingual Buprenorphine: Participants randomized to this arm will be provided SLB daily maintenance per usual Opioid Treatment Programs protocols in-jail. Post-release, SLB arm participants will receive follow-up care at Bellevue Hospital’s Addiction Medicine or at non-BHC/NYU community providers, per their preference. We expect and will encourage most participants to follow-up at Bellevue, though this is not mandated. Typically an initial week's supply of SLB is prescribed within one week of release. At visit 2, week 2, SLB participants typically refill a two week's supply of daily maintenance SLB. SLB participants will continue to receive 2-4 week refill prescriptions for SLB from visit 2, week 2 through visit 6, week 8. Regardless of SLB prescribing patterns, they are encouraged to attend study follow-up visits at BHC per the same study schedule (Weeks 1,2,4,6,8). After the completion of Visit 5 Week 8, SLB participants will be offered XRB treatment in the 24 week therapeutic extension. If interested, participants would receive one or more XRB injections during the

period of Weeks 8, 12, 16, 20, 24, and 28 followed by a final safety documentation visit at Week 32.

For both treatment arms, urine toxicology tests will be conducted at all follow-up visits at the BHC Addiction Medicine Clinic in order to detect any illicit substance use as well as buprenorphine adherence.

STUDY SCHEDULE

**Test/Procedures**	Screening/ Randomization	Treatment Period &Follow-Up					XRB 20 Week Treatment & Follow-Up					
**Study Visit Number**	**0**	**1**	**2**	**3**	**4**	**5**	**6**	**7**	**8**	**9**	**10**	**11**
**Week**	0	1	2	4	6	8	12	16	20	24	28	32
**Informed Consent** [1]	X											
**HIPAA** [2]	X											
**Locator Form**	X											
**Medical History**	X											
**Psychological History**	X											
**Physician Progress Note**	X	X	X	X	X	X	X	X	X	X	X	X
**Confirm Eligibility**	X											
**Randomization**	X											
**Adverse Event Forms (AE, SAE, BUP specific AE)**		X	X	X	X	X	X	X	X	X	X	X
**Clinical Assessment of Intervention** [3]						X						X
**Urine Toxicology**		X	X	X	X	X	X	X	X	X	X	X
**Pregnancy Test**				X		X		X			X	
**Demographics**	X											
**MOUD History/Preference**	X											
**ASI-Modified Lifetime/CJS-Baseline**	X											
**Timeline Followback**	X	X	X	X	X	X	X	X	X	X	X	X
**Inmate Lookup Form**[4]		X	X	X	X	X						
**New Arrests and Days Incarcerated Form**[4]		X	X	X	X	X						
**Overdose Form**		X	X	X	X	X	X	X	X	X	X	X
**XR-B Administration Form**	X			X			X	X	X	X	X	

**NYS Prescription Monitoring Data (SLB- Only)**		X	X	X	X	X						
**Dispense Study Medications[5]**	X, #	X, #	X, #	X, #	X, #	X	X	X				
**Treatment Retention**		X	X	X	X	X						
**Open-Ended Interviews[6]**		X				X						
**Qualitative Interviews[7]**						X		X	X		X	
**Treatment Satisfaction Form**						X						X

Annotations:

1. All patients must sign an informed consent consistent with ICH-GCP guidelines prior to participation in this trial, which includes performing any screening procedures, medication washout and any restrictions.
2. HIPAA authorization must be obtained on all patients participating in the study at Visit1.
3. Clinical Assessment must be completed per protocol instructions and by the treating investigator for that specific patient.
4. These instruments will be available for each visit, however they may/may not be filled out depending on participant's interaction with CJS
5. X = XRB, # = SLB; dispensation schedule varies depending on release date
6. Open-Ended interview should occur at participant's first & final community visit
7. For eligible XRB participants only, occurs at week 8 and final community visit.

6.3.2 Informed Consent

Potential participants interested in buprenorphine maintenance (currently a NYC jail standard of care) will be offered study information and encouraged to enroll. The study team will receive IRB-approval for all study documents, informational handouts, informed consent forms, informed consent quizzes, and all CRFs prior to screening and randomizing participants. Consent forms describing in detail the study agent, study procedures, and risks are given to the participant and written documentation of informed consent is required prior to starting intervention/administering study product. Written informed consent including a consent quiz adapted from the parent grant protocol will be used to document informed consent. Participants will have the opportunity to carefully review the written consent form and ask questions prior to signing. The participants may withdraw consent at any time throughout the course of the trial. A copy of the signed informed consent document will be given to the participants for their records. The rights and welfare of the participants will be protected by emphasizing to them that the quality of their medical care will not be adversely affected if they decline to participate in this study.

A copy of the signed informed consent document will be stored in the subject's research record. The consent process, including the name of the individual obtaining consent, will be thoroughly documented in the subject's research record. Any alteration to the standard consent process (e.g. use of a translator, consent from a legally authorized representative, consent document presented orally, etc.) and the justification for such alteration will likewise be documented.

6.3.3 Screening

Potential participants will meet with study staff to learn about study procedures, buprenorphine (XRB and SLB) treatment, and follow-up protocols. A pre-screen checklist will evaluate eligibility. All adult jail inmates with an upcoming release date, current opioid use disorder diagnosis, and currently maintained on sublingual buprenorphine-naloxone will be potential participants. If interested and eligible, participants will complete the informed consent process (ICF and ICF quiz). Once informed consent is obtained, baseline assessments will be administered prior to randomization. The baseline assessments include a drug and alcohol use history (ASI-Lite), demographics, a pre-arrest recall of drug and alcohol use (Timeline Followback), prior medication treatment for opioid use disorder exposure and preferences (MOUD Preference form), and jail Electronic Medical Record medical and psychiatric history and laboratory data, including HIV and HCV status.

6.3.4 Recruitment, Enrollment and Retention

Recruitment: Adult inmates with known Department of Corrections release dates and recent or on-going opioid treatment are housed in two NYC jail facilities, both on Rikers Island; the Eric M. Taylor Center (male) and the Rose M Singer Center (female). Approximately 150 persons a month leave this facility with an opioid use disorder diagnosis and on active OUD treatment (e.g. buprenorphine, methadone, naltrexone, or counseling). NYULMC study staff with NYC Health+Hospitals clinical credentials and HHC-approved access to Rikers electronic medical record database (EMR) will search for opioid dependent diagnoses, in-jail opioid treatment program participation and pending release dates of potential participants

located in this housing unit. Potential participants interested in buprenorphine maintenance (currently a NYC jail standard of care) will be offered study information and encouraged to enroll. As current daily maintenance of sublingual buprenorphine is required in order to be eligible for this pilot, participants will be already be affiliate with the in-jail Opioid Treatment Program and will be referred by the Opioid Treatment Program staff to Study Staff for further education about the trial and to schedule written informed consent and a comprehensive screening visit. Monetary compensation for time and travel are intended to facilitate and encourage follow-up visit attendance; transportation services for follow-up visits are otherwise not provided.

Informed Consent and Enrollment/Randomization: Study staff will obtain informed consent via the completion of the informed consent form and informed consent quiz. Consented and eligible participants will be randomized by sealed envelope or a web-based randomizer 1:1, XRB vs. SLB. Medications will be dispensed at the screening/randomization visit in-jail (visit 1, week 0) for both arms. All follow-up visits (visit 1-6) will take place at Bellevue Hospital Center's Addiction Medicine clinic and medication (XRB) will be provided free-of-charge.

6.3.5 On Study Visits

Screening and Randomization (~1.5 hours):

- Informed Consent & Informed Consent Quiz
- Baseline assessments:
    - Drug and alcohol use history (ASI-Lite)
    - Pre-arrest recall of drug and alcohol use (Timeline Followback)
    - Prior Medication Treatment for Opioid Use Disorder exposure & preferences (MOUD Preference form)
    - Jail Electronic Medical Record medical and psychiatric history & laboratory data, including HIV and HCV status
- Randomization envelope opened & completed by study staff and participant
    - Medication dispensed (XRB or SLB)
    - *Additional in-jail injections of XRB may occur if participant is incarcerated for over 4 weeks.*

Follow-Up Visits (v1-4, ~1 hour)

- Administrative data for SLB medication refills and post-release XRB injections
- Self-reported opioid and other drug use (Timeline Followback)
- Urine specimen collection and toxicology testing
- Adverse Event and Serious Adverse Event monitoring (SAFTEE)
- NYS Prescription Monitoring Data for community SLB pharmacy refill reporting
    - I-STOP/PMP Registry
- Treatment Retention Form completed
- *At visit 1 week 1, open-ended interview will be administered.*

- *At visit 3 week 4, second XRB administered, depending on prior injection date.*
- *At visit 3 week 4, pregnancy test will be administered..*

Research Visit 5 Week 8 (Final Visit, ~1 hour):

- Medication is not dispensed
- For participants electing to continue in the 24 week extension, initial and/or additional XRB injection could be administered at this time point
- Self-reported opioid and other drug use (Timeline Followback)
- Urine specimen collection and toxicology testing
- Pregnancy testing
- Treatment Retention completed
- Treatment Satisfaction completed
- Adverse Event and Serious Adverse Event monitoring (SAFTEE)
- NYS Prescription Monitoring Data for community SLB pharmacy refill reporting
  - I-STOP/PMP Registry
- Open-ended interview with participants concerning XRB vs. SLB treatment satisfaction and ease-of-use

Treatment Visit 6-10 Week 12,16, 20, 24, 28 (~1 hour):

- 24 Week Extension Participants Only:
  - Self-reported opioid and other drug use (Timeline Followback)
  - Urine specimen collection and toxicology testing
  - Adverse Event and Serious Adverse Event monitoring (SAFTEE)
  - *XRB administered, depending on prior injection date.*
  - *At visit 7 week 16 and visit 10 week 28, pregnancy test will be administered*
  - *Qualitative Interviews (randomized to XRB treatment arm only) will be conducted at XRB injection treatment visits depending on participants' medication schedules.*

Safety Visit 11 Week 32 (~1 hour, final visit):

  - Self-reported opioid and other drug use (Timeline Followback)
  - Adverse Event and Serious Adverse Event monitoring (SAFTEE)
  - Qualitative Interviews if applicable
  - Treatment Satisfaction form

In-Jail Feasibility:

- Assessments of XRB vs. SLB
    - Logging the % eligible enrolling in the study
    - The density of medical/nursing/pharmacy visits per arm and per participant
    - Rates of medication diversion.

6.3.6 End of Study and Follow-up

After completion of the final visit (Visit 5, Week 8), study participation is complete for all participants who do not elect to continue and/or initiate treatment in the 24 week therapeutic extension of XRB treatment. All participants will be provided information on XRB and SLB and will be encouraged to continue treatment with the community opioid treatment provider of their choice. All participants will have the option of accessing services at Bellevue, including buprenorphine and methadone maintenance treatment. It is likely that the majority of participants in both arms will continue on SLB maintenance with the BHC Addiction Medicine clinic. XRB may become a usual care option at BHC during the course of this trial, depending on NYS Medicaid policies and H+H formulary updates.

For participants who continue in the 24 week therapeutic extension, after completion of the final safety visit (Visit 11, Week 32), study participation is complete. All participants will be provided the same information as highlighted above. It is likely that the majority of participants in this arm will continue on SLB maintenance with the BHC Addiction Medicine clinic.

In-jail feasibility will be assessed during and post study completion as the data is made available. All adverse event and serious adverse event monitoring (SAFTEE) will take place throughout the study and will be documented/reported as necessary.

6.3.7 Removal of subjects

Participation in this pilot, proof-of-concept, RCT is entirely voluntary and participants are free to withdraw at any time. In the event of early study termination, study staff will complete the study termination form, modeled from a CRF of the parent XOR study. Any participant withdrawing from the study prior to full study completion, regardless of study group, stands a risk of relapsing to illicit opioid or other drug/alcohol misuse. The study will assist with treatment referrals for detox services at Bellevue Hospital Center, but will not otherwise directly provide such services or medications. Otherwise any XRB or SLB patient who has relapsed and is not interested in continuing their baseline study condition (i.e., an XRB participant does not wish to continue injections due to side effects or for any other reason) will be encouraged to pursue other appropriate community treatment, the menu of which includes the robust addiction service offerings at Bellevue Hospital Center (BHC). BHC's services are available to all persons, regardless of insurance status or an ability to pay, and include emergency detox inpatient services, methadone treatment, office-based buprenorphine, and intensive outpatient and dual diagnosis programs. These services will consist of usual care occurring outside of the study and will not be directly provided by or paid for by the study.

6.4.1 Statistical Design

This pilot study is exploratory, and Aim 1 is a qualitative estimation of the feasibility and acceptability of this new form of buprenorphine in a large urban jail setting. The study will be moderately well-powered using a superiority contrast (A>>B) of XRB vs. SLB surrounding Aim 2, retention in treatment post-release. Current rates of successful retention in community buprenorphine treatment immediately at one month post-release are 30% or lower (6). This current administrative data reflects closely results seen in an original BUP vs. methadone NYC jail RCT conducted in 2006-2008 (7). If XRB retention at one month post-release (Week 4) is 70% or greater, the study would be powered to detect a significant difference between arms. Analysis of treatment retention among XRB vs. SLB will consist of simple 2x2 tables for counts and proportions of weeks retained in treatment by arm. We are unlikely to extensively model and control for confounders or interaction terms.

6.4.2 Sample Size Considerations

A total sample size of N=50 would allow for an estimate of an Odds Ratio of 2.0-3.0 for the rate of persons successfully in community buprenorphine treatment at 4 weeks post-release (71% vs. 30% success favoring XRB; two-sided alpha of 0.05; 80+% power). The proposed sample size will not provide a definitive test of intervention efficacy, but is sized for feasibility testing.

6.4.3 Handling of Missing Data

Follow-up among individual participants at Bellevue Hospital is encouraged with XOR's monetary incentives and tracking protocols. Participants in either arm choosing buprenorphine f/u at Bellevue will be particularly likely to complete follow-up. Administrative and program data covering community pharmacy fills of buprenorphine products and community provider reporting will be used to supplement treatment retention outcomes (Aim 2). Missing opioid urine toxicology data is considered MissingAsPositive.

7 - Trial Administration

7.1 Ethical Considerations

Federal Research among Prisoners. See section 5.3.2.

Emotional Discomfort: There is a small chance that participants may become upset when discussing their history of addiction problems, criminal justice involvement, family conflict, prior trauma, or role failure, etc. We will discontinue administration of research instruments if a subject shows great discomfort or asks to terminate an interview. Such events have not been observed in our preliminary studies.

7.2 Institutional Review Board (IRB) Review

The protocol, informed consent form(s), recruitment materials, and all participant materials will be submitted to the appropriate IRB and institutional research committees for review and approval. Approval of both the protocol and the consent form must be obtained before any participant is enrolled. Any amendment to the protocol will require review and approval by the IRB before the changes are implemented to the study. All changes to the consent form will be IRB approved; a determination will be made regarding whether previously consented participants need to be re-consented.

7.3 Subject Confidentiality

Participants will be asked to provide information regarding a number of sensitive behaviors (e.g., alcohol and drug use, sexual history, criminal history and on-going illicit activities). This type of personal information divulged by participants at study visits may have adverse social and other unknown consequences for participants if released. Therefore, in addition to the safeguards put in place for the collection and storage of all data as described above (section 11.0 Data Collection and Management), the study team obtained a Federal Certificate of Confidentiality (COC #DA-13-154) to encompass protocol activity and further safeguard the possible risk of released confidential information. We will provide all staff with training in their responsibilities for maintaining subject confidentiality; we will use unique identifiers to identify subjects in the database; all data will be kept in locked filing cabinets or on our secure server to which only the investigators and project manager will have access to. Study findings will utilize only aggregate data and no publication or presentation will involve any use of individual information.

New York City's H+HC's Human Subject's Research Protections Programs Policies and Procedures procedure #26.3 states:

If a patient is taking part in a Research Project involving a drug, device, or procedure (therapeutic trial), the patient's participation must be clearly noted in the patient's electronic medical record. Researchers should scan and upload Informed Consent forms into the electronic medical record when and where possible, preferably to a research folder. Research Records related to an FDA application must be maintained in accordance with FDA requirements.

Therefore, if you are randomized to XRB: Information about your participation in this study will be entered into your Electronic Medical Record (EMR). Once placed in your EMR, the information will be available to all of your providers who participate in the EMR system at NYC Health + Hospitals including those at Bellevue Medical Records office (HIM). The purpose of this entry is to provide research information that has the potential to negatively impact your medical care. Bellevue HIM will scan and upload your informed consent to your EMR, and are bound by the rules of confidentiality not to reveal your identity to others.

7.4 Deviations/Unanticipated Problems

Protocol Deviations: A protocol deviation is any noncompliance with the clinical trial protocol, GCP, or Manual of Procedures (MOP) requirements. The noncompliance may be either on the part of the participant, the investigator, or the study site staff. As a result of deviations, corrective actions are to be developed by the site and implemented promptly.

These practices are consistent with ICH E6:

- 4.5 Compliance with Protocol, sections 4.5.1, 4.5.2, and 4.5.3
- 5.1 Quality Assurance and Quality Control, section 5.1.1
- 5.20 Noncompliance, sections 5.20.1, and 5.20.2.

It is the responsibility of the site PI/study staff to use continuous vigilance to identify and report deviations within 30 working days of identification of the protocol deviation, or within 30working days of the scheduled protocol-required activity.

Protocol deviations must be reported to the local IRB per their guidelines. The site PI/study staff is responsible for knowing and adhering to their IRB requirements. Further details about the handling of protocol deviations will be included in the MOP.

Unanticipated Problems: Incidents or events that meet the OHRP criteria for UPs require the creation and completion of an UP report form. It is the site investigator's responsibility to report UPs to their IRB and to the DCC/study sponsor. The UP report will include the following information:

- Protocol identifying information: protocol title and number, PI's name, and the IRB project number;
- A detailed description of the event, incident, experience, or outcome;
- An explanation of the basis for determining that the event, incident, experience, or outcome represents an UP;
- A description of any changes to the protocol or other corrective actions that have been taken or are proposed in response to the UP.

To satisfy the requirement for prompt reporting, UPs will be reported using the following timeline:

UPs that are SAEs will be reported to the IRB and to the DCC/study sponsor within 30 days of the investigator becoming aware of the event. Any other UP will be reported to the IRB

and to the DCC/study sponsor within 30 days of the investigator becoming aware of the problem. All UPs should be reported to appropriate institutional officials (as required by an institution's written reporting procedures), the supporting agency head (or designee), and OHRP within 30 days of the IR's receipt of the report of the problem from the investigator.

7.5 Data Quality Assurance

Study clinicians and research staff will undergo the same baseline training at the inception of the study. The Program/Project Manager and Data Management staff will ensure the quality of the clinicians' and the research assistants' administration of study assessments and instruments and of integrity of the data recorded through regular reviews and on-going data monitoring.

7.5.1 Data Collection

Electronic case report forms (CRFs) will be used to manage the data for this study, and the Project Manager will work with study personnel to ensure the completeness and integrity of all study data. These electronic CRFs will be taken from the parent XOR U01 NIDA study. All data will be checked in real time, stored in a centralized database, reviewed and monitored for completeness and accuracy, and undergo a final cleaning following the last subject visit, following which the study database will be locked. REDCap will be the data entry and database platform for this study.

Protected Health Information (PHI) will be collected and stored in the form of each participant's original signed informed consent and locator form. These written documents will be filed as individual charts and locked securely in a private Department of Population Health office cabinet. These charts will be accessed to call and follow-up with patients post-release and to maintain the ICF on file. Participants will be assigned sequentially numbered unique study ID numbers at the time of consent (e.g. #001-50). These ID numbers will be the only identifier entered on Case Report Forms (CRF) and research assessments to distinguish one participant from another. Secure laptops will be used for web-based data entry, CRF variables will be input into a secure central database on an NYULMC server. The laptops themselves will be password-protected, but will not store PHI, study ID number information or any research assessment data and will be used for web-based data entry only. To maintain a link between the study ID and PHI for purposes of follow-up and record-keeping, individual participant charts and research assessment/CRF data there will be a master participant ID key that will contain each participant's PHI (name, DOB) and their corresponding unique study ID number. This master key identifier file (.xls) will be maintained by the PI and Project Manager on a secure NYULMC desktop file and accessible only to study staff.

At the end of a three-year period following study closure, written identifiable data will be destroyed. De-identified study data will remain in digital and written file storage for a period of 6 years following study conclusion and protocol close per standard NYU IRB guidelines. The final de-identifiable digital dataset may be used by the Principal Investigator or Co-Investigator for secondary analysis, in which case future IRB-approval would be sought.

7.5.1.1 Access to Source

Source Documents (PHI):

- Original signed informed consent and consent form quiz
- Participant locator form
- Randomization Form
- Consent for the release of confidential alcohol or drug treatment information
- Consent for the audio recording of in depth qualitative interviews

These source documents containing PHI will be filed as individual charts and locked securely in a private Department of Population Health office cabinet. These charts will be accessed to call and follow-up with patients post-release and to maintain the ICF on file.

Source Documents (Case Report Forms, De-Identified):

- ASILite: Baseline and Follow-up
- Demographics: Baseline
- Pre-arrest recall of drug and alcohol use (Timeline Followback)
- Self-report drug and alcohol use, post-release (Timeline Followback)
- Medication Treatment for Opioid Use Disorder exposure/preference (MOUD Preference)
- Treatment Retention Form
- Open-Ended Interview Form
- Qualitative Interview Form
- Medical and Psychiatric History & Laboratory data (pulled from EMR)
- NYS Prescription Monitoring Form
- New Arrests & New Incarceration Form
- Inmate Lookup Form
- Urine Toxicology Report
- Pregnancy Testing
- Adverse Event and Serious Adverse Event reporting

Participants will be assigned sequentially numbered unique study ID numbers at the time of consent (e.g. #001-50). These ID numbers will be the only identifier entered on Case Report Forms (CRF) and research assessments to distinguish one participant from another. Secure laptops will be used for web-based data entry, CRF variables will be input into a secure central database on an NYULMC server. The laptops themselves will be password-protected, but will not store PHI, study ID number information or any research assessment data and will be used for web-based data entry only. To maintain a link between the study ID and PHI for purposes of follow-up and record-keeping, individual participant charts and research assessment/CRF data there will be a master participant ID key that will contain

each participant's PHI (name, DOB) and their corresponding unique study ID number. This master key identifier file (.xls) will be maintained by the PI and Project Manager on a secure NYULMC desktop file and accessible only to study staff.

7.5.1.2 Data Storage/Security

Data will be collected on both hard-copy paper CRFs and electronic CRFs. Protected Health Information (PHI) will be collected and stored in the form of each participant's original signed informed consent, informed consent quiz, randomization form, and locator form. These written documents will be filed as individual charts and locked securely in a private Department of Population Health office cabinet. These charts will be accessed to call and follow-up with patients post-release and to maintain the ICF on file. Participants will be assigned sequentially numbered unique study ID numbers at the time of consent (e.g. #001-50). These ID numbers will be the only identifier entered on Case Report Forms (CRF) and research assessments to distinguish one participant from another. Secure laptops will be used for web-based data entry, CRF variables will be input into a secure central database on an NYULMC server. The laptops themselves will be password-protected, but will not store PHI, study ID number information or any research assessment data and will be used for web-based data entry only. To maintain a link between the study ID and PHI for purposes of follow-up and record-keeping, individual participant charts and research assessment/CRF data there will be a master participant ID key that will contain each participant's PHI (name, DOB) and their corresponding unique study ID number. This master key identifier file (.xls) will be maintained by the PI and Project Manager on a secure NYULMC desktop file and accessible only to study staff. All hard copy CRFs labeled only with ID numbers will be filed in subject files, numbered sequentially. These subject files will be kept in a securely locked, private Department of Population Health office cabinet, separate from the individual locator charts.

7.6 Study Records

Study Records will include:

- All regulatory documents, kept up-to-date in regulatory binders and online Research Navigator
- All IRB approved protocol versions
- All IRB approved informed consent forms
- All IRB approved case report forms including surveys
- Individual participant charts
- Original signed ICF & ICF quiz
- Randomization form
- Locator Form
- Medication information
- Subject ID File
- All completed CRFs & surveys, organized by treatment visit.

7.6.1 Retention of Records

At the end of a three-year period following study closure, written identifiable data will be destroyed. De-identified study data will remain in digital and written file storage for a period of 6 years following study conclusion and protocol close per standard NYU IRB guidelines. The final de-identifiable digital dataset may be used by the Principal Investigator or Co-Investigator for secondary analysis, in which case future IRB-approval would be sought.

7.7 Study Monitoring

The PI, Project/Program Manager, and study staff will work to ensure that all information collected is accurate and properly protected/secured. The Principal Investigator, Dr. Joshua D. Lee, will assume responsibility for monitoring of data collection and of participant safety. Any serious or unexpected adverse event will be reported immediately to: 1) the NYC DOHMH and NYUSOM-IRBs.

7.8 Data Safety Monitoring Plan

A DSMB will monitor this trial. Dr. Rotrosen (NYU Psychiatry) will chair the DSMB. Two other board members are to be determined. The DSMB will conduct ongoing protocol review, including data, protocol compliance, safety and efficacy data, in compliance with NIDA and NYU IRB guidelines. All board members will meet NIDA requirements regarding background and experience, and none will have ethical conflicts, including financial interest related to study outcome. Individuals invited to serve on the board will disclose any potential conflicts in writing. The board will meet every six months (unless more frequent meetings are deemed necessary. Dr. Lee and other research personnel will open each meeting with a report on the trial's status, followed by a closed session under the direction of the DSMB chairperson, during which time the investigators and research team may be present. This will be followed by an executive session restricted to DSMB members. Issues related to subject safety, conflict of interest, confidentiality, and ongoing study review (including AEs, SAEs, and regulatory issues) will be assessed. Following each DSM Board meeting, recommendations will be made to Dr. Lee, and a final report (edited by all Board members) will be prepared for reporting to NIDA, the DOHMH and NYU IRBs. The Data Safety Monitoring Plan (DSMP) includes stopping rules that specify the outcome differences detected between groups during an interim analysis that can result in stopping the pilot trial. In general, stopping rules will reflect one of the following conditions: 1) there is clear evidence of harm or harmful side-effects of the treatment; 2) there is not likelihood of demonstrating treatment benefit; 3) there is overwhelming evidence of the benefit of the treatment. However because we are comparing alternative paradigms involving a study medication (XRB) or community treatment as usual (SLB) that are already FDA-approved as opioid treatment and do not suggest significant safety considerations, early stopping on the basis of clear benefit (yes/no) is not anticipated in this trial.

7.9 Study Modification

Study modifications, such as the addition of new study personnel, may occur throughout this pilot study. All modifications will be submitted to the NYU IRB for review. All IRB approved modifications will be stored in regulatory binders. If the IRB determines changes to the protocol must be made, the protocol will be edited, submitted for NYU IRB review, and all updated versions will then be implemented going forward.

7.10 Study Discontinuation

There is very little chance that this pilot will be discontinued as both investigational medications have been FDA approved and have been shown to be safe & effective. Additionally, this pilot, proof-of-concept, RCT has been approved by NIDA for the period of 1 year only. Study termination may be possible in the event of overwhelmingly significant efficacy differences between groups or unacceptable adverse events.

7.11 Study Completion

The study has an estimated completion date of June 29, 2019.

7.12 Conflict of Interest Policy

The independence of this pilot from any actual or perceived influence, such as by the pharmaceutical industry, is critical. Therefore any actual conflict of interest of persons who have a role in the design, conduct, analysis, publication, or any aspect of this trial will be disclosed and managed. Furthermore, persons who have a perceived conflict of interest will be required to have such conflicts managed in a way that is appropriate to their participation in the trial. The study leadership in conjunction with the NYU IRB has established policies and procedures for all study group members to disclose all conflicts of interest and will establish a mechanism for the management of all reported dualities of interest.

Any investigator who has a conflict of interest with this study (patent ownership, royalties, or financial gain greater than the minimum allowable by their institution, etc.) must have the conflict reviewed by the NYU Langone Conflict of Interest Management Unit (CIMU) with a Committee-sanctioned conflict management plan that has been reviewed and approved by the study sponsor prior to participation in this study. All NYULMC investigators will follow the applicable conflict of interest policies.

7.13 Funding Source

This pilot is supported by a NIDA Administrative Supplement as part of the on-going XOR U01 award.

Appendices

Appendix #	Name	Title	Section	Topic
1	demographics_somatics_xor_nyu_v4.0.pdf		6 Methods	6.2.1 Efficacy
2	addiction_severity_index-lite_baseline_8.07.2014.pdf		6 Methods	6.2.1 Efficacy
3	addiction_severity_index-lite_drug_history_8.01.2014.pdf		6 Methods	6.2.1 Efficacy
4	addiction_severity_index-lite_follow-up_7.25.2014.pdf		6 Methods	6.2.1 Efficacy
5	tlfb_pre-arrest_calendar.pdf		6 Methods	6.2.1 Efficacy
6	tlfb_somatics_xor_nyu_v2_0.pdf		6 Methods	6.2.1 Efficacy
7	urine_toxicology_nyu_xor_v2.0.pdf		6 Methods	6.2.1 Efficacy
8	adverse_event_somatics_xor_nyu_v2.0.pdf		6 Methods	6.2.2.1 AE Definition and Reporting
9	sae_xor_nyu_v_1.0.pdf		6 Methods	6.2.2.1 AE Definition and Reporting

List of Tables

Title
Study Visit Schedule
Study Flowchart

References

1. The National Alliance of Advocates for Buprenorphine Treatment. What exactly is Buprenorphine (2018, June 12); Retrieved from: https://www.naabt.org/faq_answers.cfm?ID=2.

2. Indivior. FDA Approves SUBLOCADE™ (Buprenorphine Extended-Release), the First and Only Once-Monthly Injectable Buprenorphine Formulation to Treat Moderate to Severe Opioid Use Disorder. 2017;

3. CDC. Drug Overdose Death Data. 2017;

4. CDC. Wide-ranging online data for epidemiologic research (WONDER). 2017;

5. Alex B, Weiss DB, Kaba F, Rosner Z, Lee D, Lim S, Venters H, MacDonald R. Death After Jail Release. Jan 2017; 23(1):83-87.

6. Giftos, J. Personal Communication, 2/1/2018. Medical Director, Opioid Treatment Program.

7. Magura S, Lee JD, Hershberger J, Joseph H, Marsch L, Shropshire C, Rosenblum A. Buprenorphine and methadone maintenance in jail and post-release: a randomized clinical trial. Jan 1, 2009; 99(1-3):222-230.

8. Lee JD, Grossman E, Truncali A, Rotrosen J, Rosenblum A, Magura S, Gourevitch MN. Buprenorphine-naloxone maintenance following release from jail. 2012; 33(1):40-47.

9. Brinkley-Rubinstein L, Zaller N, Martino S, Cloud DH, McCauley E, Heise A, Seal D. Criminal justice continuum for opioid users at risk of overdose. Feb 24, 2018; doi: S0306-4603(18)30089-3.
