# Supplementary material for: Comparison of Treatment Retention of Adults With Opioid Addiction Managed With Extended-Release Buprenorphine vs Daily Sublingual Buprenorphine-Naloxone at Time of Release From Jail
Source: JAMA Netw Open. 2021 Sep 8;4(9):e2123032. doi: 10.1001/jamanetworkopen.2021.23032 (PMC8427378; doi:10.1001/jamanetworkopen.2021.23032)
Supplement: Supplement 2. — Data Sharing Statement [file jamanetwopen-e2123032-s002.pdf]

Lee JD, Malone M, McDonald RD, et al. Comparison of treatment retention of adults with opioid addiction managed with extended-release buprenorphine vs daily sublingual buprenorphine-naloxone at time of release from jail. *JAMA Netw Open*. 2021;4(9):e2123032. doi:10.1001/jamanetworkopen.2021.23032

Data Sharing Statement

Data

Data available: Yes
Data types: Deidentified participant data, Data dictionary
How to access data: Joshua D. Lee; joshua.lee@nyulangone.org
When available: With publication

Supporting Documents

Document types: None

Additional Information

Who can access the data: Researchers whose proposed use of the data has been approved.
Types of analyses: For a specified purpose.
Mechanisms of data availability: With investigator support and after approval of a proposal.
Any additional restrictions: N/A
